# Carboxylated Acyclonucleosides: Synthesis and RNase A Inhibition

**DOI:** 10.3390/molecules20045924

**Published:** 2015-04-03

**Authors:** Kaustav Chakraborty, Swagata Dasgupta, Tanmaya Pathak

**Affiliations:** Department of Chemistry, Indian Institute of Technology Kharagpur, Kharagpur 721302, India; E-Mail: kaustav@chem.iitkgp.ernet.in

**Keywords:** acyclonucleosides, RNase A, inhibition, kinetics, docking

## Abstract

Strategically designed carboxylated acyclonucleosides have been probed as a new class of RNase A inhibitors. Several experimental and theoretical studies have been performed to compile relevant qualitative and quantitative information regarding the nature and extent of inhibition. The inhibition constant (*K_i_*) values were determined using a UV-based kinetics experiment. The changes in the secondary structure of the enzyme upon binding with the inhibitors were obtained from circular dichroism studies. The binding constants for enzyme-inhibitor interactions were determined with the help of fluorescence spectroscopy. Docking studies were performed to reveal the possible binding sites of the inhibitors within the enzyme. The cytosine analogues were found to possess better inhibitory properties in comparison to the corresponding uracil derivatives. An increment in the number of carboxylic acid groups (-COOH) in the inhibitor backbone was found to result in better inhibition.

## 1. Introduction

Ribonucleases are a family of digestive enzymes that degrade RNA [1]. Effective inhibition of their enzymatic activity has become a topic of growing interest with the realization that the detrimental biological activities manifested by certain members of this family [2,3,4,5,6] are critically dependent upon their ribonucleolytic activity [7,8]. Ribonuclease A (RNase A) is a representative member of this family [9,10] that works at the juncture of the transcription and translation processes, thereby maintaining the cellular RNA levels. Structural homology among the various members of this family [11,12,13] permits the use of Ribonuclease A (RNase A) as a model system to explore structure–activity relationships.

**Figure 1:**
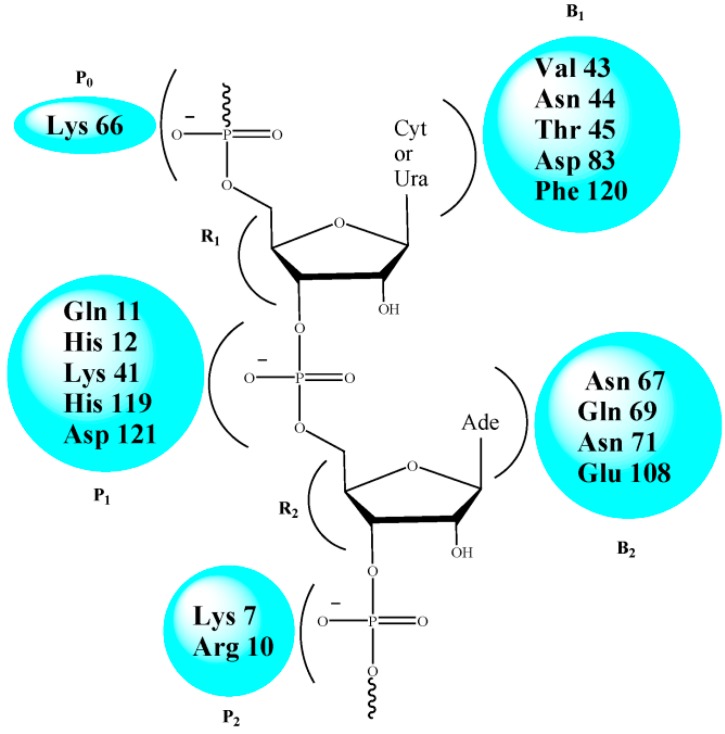
Key residues of the active site of RNase A.

Recent reports from our laboratory have revealed that modified nucleoside carboxylic acids manifest RNase A inhibitory properties in a competitive, reversible manner [14,15,16,17,18,19,20,21]. These molecules have an added advantage over the reported phosphate- or pyrophosphate-based nucleotide inhibitors [22,23,24,25,26,27,28,29,30,31] as the polyionic nature of the latter hampers their migration through the cell membrane [32]. The active site of RNase A consists of several subsites made up of polar amino acid residues for specific recognition [9,33] (Figure 1). The cleavage of the phosphodiester bond of RNA at the P_1_ subsite involves the two His residues (His 12 and His 119) participating in a conjugate acid-base mechanism [34,35]. At physiological pH, the carboxylic acid (-COOH) group(s) in the inhibitor remains deprotonated, and interacts electrostatically with the protonated His and Lys residues present at the ribonucleolytic site [36]. This perturbation of the protonating/deprotonating environment of the P_1_ subsite results in the inhibition of RNase A. 

Following the same hypothesis, it was expected that carboxylated acyclonucleosides may elicit similar inhibitory properties because of their in-built structural features. The absence of the rigid ribose ring further enhances the flexibility of these molecules, and generates additional information on the importance of the furanoside ring. We report the synthesis of several uracil- and cytosine-based modified acyclonucleosides followed by the exploration of their RNase A inhibitory properties. The nucleobases have been carefully selected, as the B_1_ subsite of RNase A shows preferential recognition towards pyrimidine bases. The acidity of the molecules has been increased via stepwise incorporation of carboxylic acid groups in the molecular framework to study the resulting effect on their inhibition capacities.

## 2. Results and Discussion

### 2.1. Synthesis of Nucleosides 

**Scheme 1:**
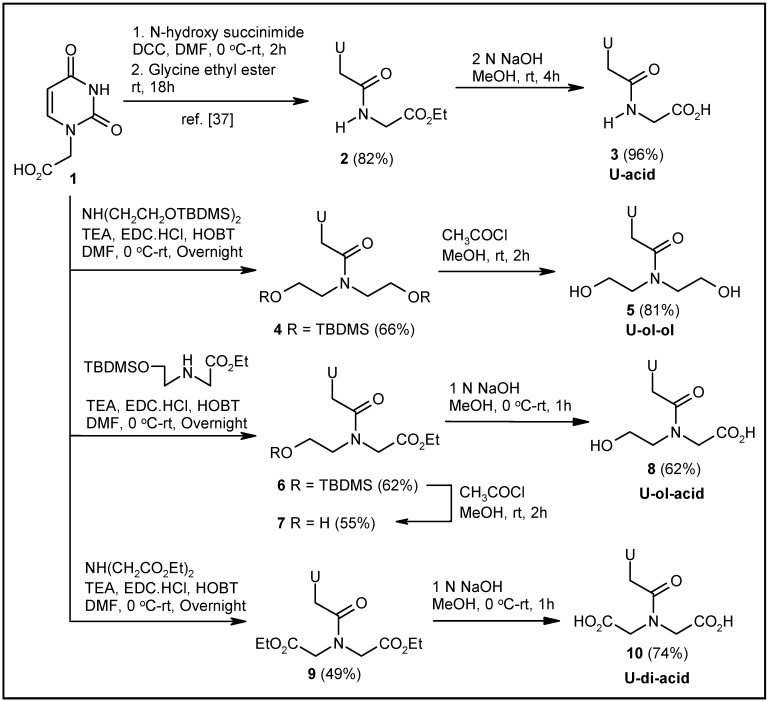
Synthesis of uracil-based modified acyclonucleosides.

The uracil-glycine conjugate **3** was synthesized according to a literature reported procedure [37] (Scheme 1). Syntheses of other modified uracil derivatives were achieved via coupling of the known uracil-1-acetic acid **1** [37] with suitable secondary amines, followed by hydrolysis and/or deprotection as required (Scheme 1). The synthesis of the uracil-diethanolamine conjugate **5** [38] was achieved by coupling **1** with the *tert*butyldimethyl silyl (TBDMS)-protected diethanolamine, and deprotecting the TBDMS groups of the coupled product **4** with a catalytic amount of CH_3_COCl in methanol (Scheme 1). TBDMS-protected N-(2-hydroxyethyl)glycine ethyl ester, on EDC-HOBT-mediated coupling with **1**, generated compound **6**. Deprotection of the TBDMS group produced compound **7**, which was converted to the corresponding acid **8** via hydrolysis (Scheme 1). Again, **1** was coupled with diethyl iminodiacetate to afford the corresponding diester **9**. The diacid derivative **10** was produced from **9** by base-mediated hydrolysis (Scheme 1). 

**Scheme 2:**
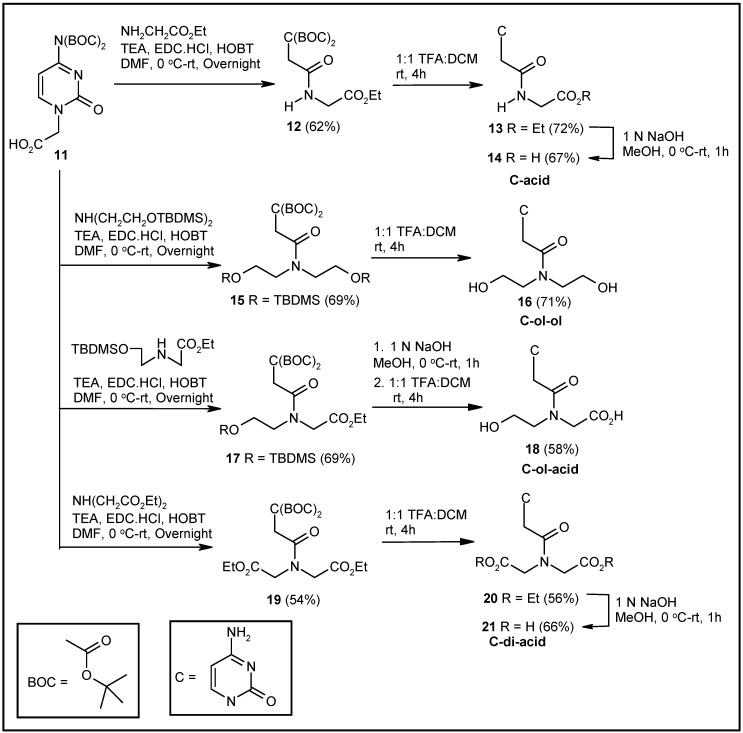
Synthesis of cytosine-based modified acyclonucleosides.

Syntheses of the corresponding cytosine-based molecules were achieved using the reported cytosine derivative **11** [39] as the starting material (Scheme 2). EDC-HOBT-mediated coupling of glycine ethyl ester to compound **11** produced the coupled product **12**. The deprotection of the *tert*butyloxy carbonyl (BOC) groups of **12** by TFA resulted in the formation of compound **13**. The hydrolysis of the ester group provided the desired acid derivative **14** (Scheme 2). Compound **11**, on coupling with TBDMS-protected diethanolamine, generated the coupled derivative **15**. TFA treatment of compound **15** afforded the deprotected cytosine-diethanolamine conjugate **16** (Scheme 2). Again, coupling of the TBDMS-protected N-(2-hydroxyethyl)glycine ethyl ester with compound **11**, followed by hydrolysis and deprotection of the resulting ester **17**, afforded the corresponding hydroxy acid derivative **18** (Scheme 2). Finally, the diacid **21** was obtained from compound **11** following a similar sequence of steps (Scheme 2). Compound **11**, on coupling with diethyl iminodiacetate, produced the coupled product **19**. Compound **19** was transformed to compound **20** by treatment with TFA. Base-mediated hydrolysis of **20** afforded the desired diacid derivative **21**.

### 2.2. RNase A Inhibition

**Figure 2:**
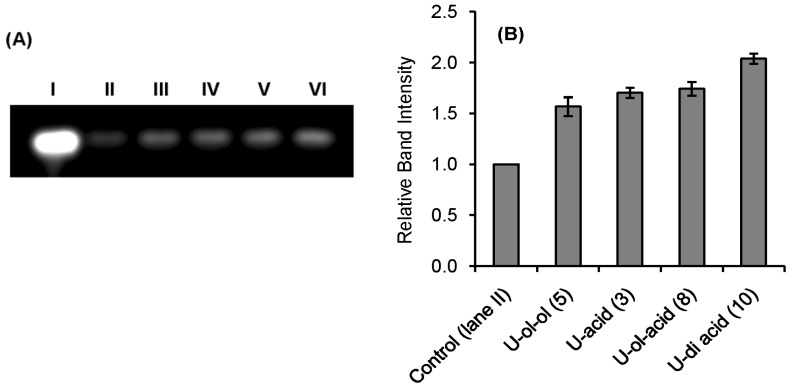
Agarose gel-based assay for RNase A (1 µM) Inhibition. (**A**) lane **I**: RNA (10 mg/mL), lane **II**: RNA + RNase A, lanes **III**, **IV**, **V**, **VI**: RNA + RNase A + **U-ol-ol** (**5**), **U-acid** (**3**), **U-ol-acid** (**8**), and **U-di-acid** (**10**) (0.5 mmol), respectively. (**B**) Histogram showing the relative band intensities of agarose gel assay (the data are the mean ± SD).

**Figure 3:**
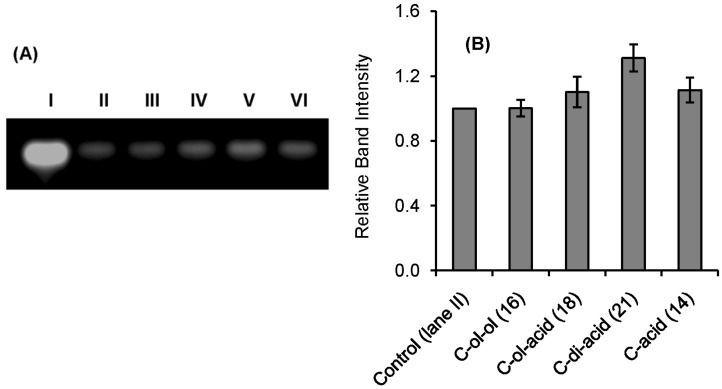
Agarose gel-based assay for RNase A (1 µM) Inhibition. (**A**) lane **I**: RNA (10 mg/mL), lane **II**: RNA + RNase A, lanes **III**, **IV**, **V**, **VI**: RNA + RNase A + **C-ol-ol** (**16**), **C-ol-acid** (**18**), **C-di-acid** (**21**), and **C-acid** (**14**) (0.5 mmol), respectively. (**B**) Histogram showing the relative band intensities of agarose gel assay (the data are the mean ± SD).

Qualitative indication of RNase A inhibition by the synthetic inhibitors was obtained from a comparative agarose gel-based assay by monitoring the degradation of RNA by RNase A (Figure 2 and Figure 3). The most intense band observed in lane **I** in each gel is due to the presence of only RNA. The faint band in lane **II** is due to the maximum possible degradation of RNA by RNase A. Different intensities of bands from lane **III** to **VI** revealed different extents of inhibition by the inhibitors at a fixed concentration (0.5 mM). The histograms obtained by plotting the relative intensities of the bands revealed that compounds **U-ol-acid** (**8**), **C-ol-acid** (**18**), **U-di-acid** (**10**), and **C-di-acid** (**21**) were relatively more potent inhibitors in comparison to the others. These experimental observations reaffirmed our assumption that increasing the acidity of the molecules leads to an increase in the inhibitory property of the inhibitor.

**Figure 4:**
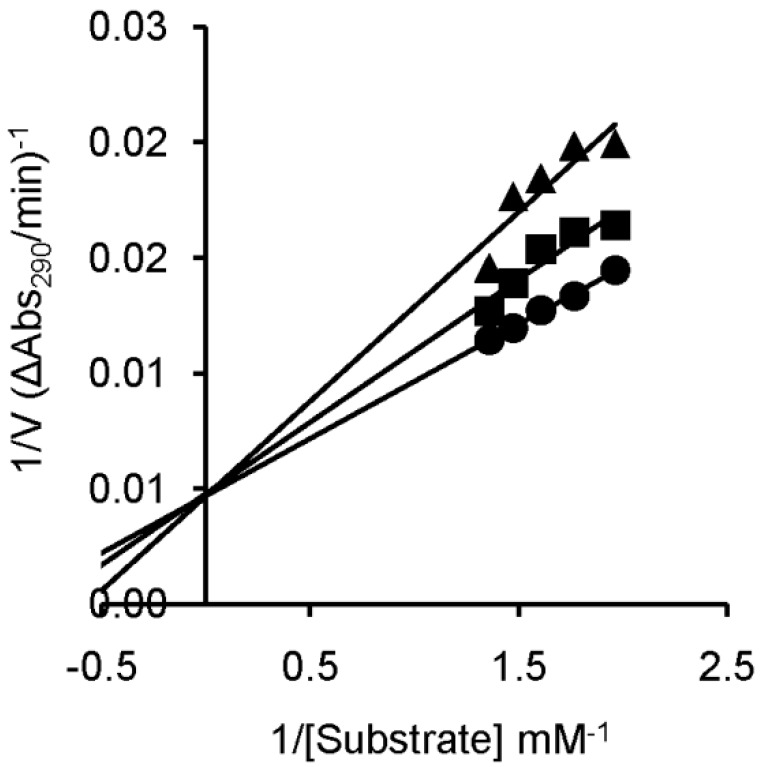
Lineweaver–Burk plot for inhibition of RNase A by **C-di-acid** (**21**) of 0.15 (▲), 0.05 (■), or 0 (●) mM, with 2',3'-cCMP concentrations of 0.75–0.52 mM and RNase A concentration of 9.8 μM.

In order to determine the type of inhibition and the inhibition constant (*K_i_*) values, kinetic experiments were performed with compounds **U-ol-acid** (**8**), **U-di-acid** (**10**), **C-ol-acid** (**18**), and **C-di-acid** (**21**). The inhibition constant values are given in Table 1. The competitive nature of inhibition in all the cases was apparent from the nature of the Lineweaver–Burk plots obtained from the kinetic experiments (Figure 4 and Figure S1 (SI)). The numerical order of the inhibition constant (*K_i_*) values indicated that **U-di-acid** (**10**) and **C-di-acid** (**21**) are the two most potent inhibitors of the series. The results are in good agreement with the results obtained from agarose gel-based assay, suggesting a correlation of inhibitory efficiency with the number of carboxylic acid groups present in the concerned inhibitor. The cytosine analogue **C-di-acid** (**21**) was found to possess superior inhibitory property in comparison to the corresponding uracil derivative **U-di-acid** (**10**).

**Table 1:** Inhibition constants (*K_i_*) of the inhibitors.

Inhibitor	*K_i_* * (μM)
**U-ol-acid** (**8**)	454 ± 9
**C-ol-acid** (**18**)	356 ± 7
**U-di-acid** (**10**)	301 ± 15
**C-di-acid** (**21**)	235 ± 9

* The data are the mean ± SD.

**Figure 5:**
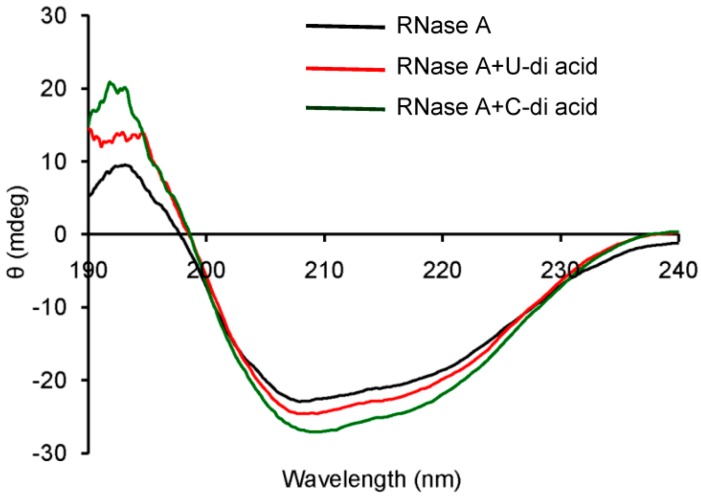
CD spectra of RNase A in absence or presence of **U-di-acid** (**10**) and **C-di-acid** (**21**).

Inhibitors of RNase A are known to perturb the secondary structure of the enzyme upon binding [40,41,42,43,44]. A model drug–protein interaction study showed that 3'-azido-3'-deoxythymidine increases the α-helix content of RNase A [40]. Similar increments were observed with 3'-*O*-carboxy esters of thymidine, which inhibited RNase A in reversible competitive mode [42]. Therefore, the probable changes in the secondary structure of the enzyme by the inhibitors were monitored by observing the CD spectra of RNase A in the absence or presence of compounds **U-di-acid** (**10**) and **C-di-acid** (**21**) (Figure 5). Both the inhibitors induced moderate changes in the secondary structure of the enzyme, which was reflected in the enhanced α-helix content. The α-helix content in native RNase A was 22.7%, which was found to increase upon binding with **U-di-acid** (**10**) and **C-di-acid** (**21**) to 24.7% and 29.2%, respectively. 

The fluorescence emission intensity of RNase A due to the presence of six Tyr residues is found to decrease upon interactions with small molecule inhibitors [40,41,42,43,44]. The emission spectrum of RNase A in presence of **U-di-acid** (**2.10**) and **C-di-acid** (**2.21**) at 25 °C showed quenching of the fluorescence intensity of Tyr residues (Figure S2, (SI)). The fluorescence quenching study was used to calculate the binding parameters for enzyme-inhibitor interactions. Binding constants (*K_b_*) calculated from the experiment were found to be in the order of 10^5^ for **C-di-acid** (**21**) and 10^4^ for **U-di-acid** (**10**) (Table S1, (SI)), indicating strong binding of the inhibitors with the enzyme.

**Figure 6:**
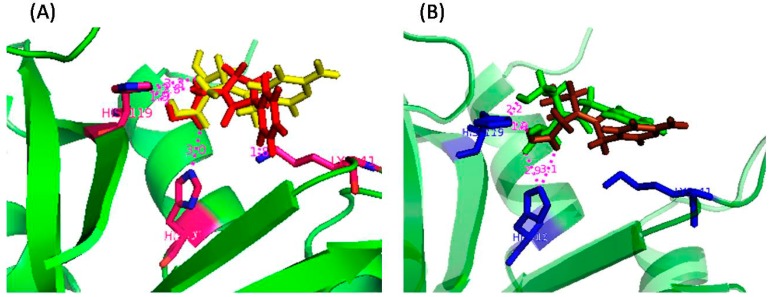
Docked poses of (**A**) **C-di-acid** (**21**) (yellow) and **U-di-acid** (**10**) (red), and (**B**) **U-ol-acid** (**8**) (green) and **C-ol-acid** (**18**) (brown) with RNase A (1FS3).

To gain an insight into the probable binding sites, protein-ligand docking studies were undertaken. The docked conformations shown in Figure 6 revealed that the nucleobase-amino acid conjugates were in close proximity to the amino acid residues of the P_1_ subsite, resulting in a competitive mode of inhibition, as observed from the kinetic study. The carboxylic acid (-COOH) groups, in each case, were positioned close to His12 and His 119, probably engaged in hydrogen bonding with the residues. For the cytosine-based inhibitors, the N3 and –NH_2_ groups of the nucleobase were near to several amino acid residues (Arg39, Lys41, Val43), thus increasing the possibility of polar interactions between them. Such interactions were absent for the uracil-derived inhibitors. These extra interactions probably contribute to the experimentally observed better inhibitory potency of the cytosine inhibitors. Apart from the interactions with the active site residues, one of the carboxylic acid (-COOH) groups of **U-di-acid** (**10**) was found to be within hydrogen bonding distance of Oε1 of Gln11. Similarly, one –COOH group of **C-di-acid** (**21**) was engaged in hydrogen bonding with Phe120. Such favorable interactions of the –COOH groups may possibly result in the better inhibition capacity of the inhibitors. The docked enzyme-inhibitor complexes also revealed that the nucleobase in the acyclic structure is not in close proximity to the amino acid residues of the pyrimidine binding subsite B_1_ (Thr45, Asp83, Phe120). It can, therefore, be assumed that the ribose ring in the nucleosides may have a role in recognition of the inhibitors by the enzyme. A detailed account of all the interactions between the inhibitors and the enzyme has been provided in Table S2 (SI).

A further clarification regarding the possible binding sites of the inhibitors was obtained by calculating the changes in accessible surface area (*ΔASA*) of the interacting residues between the free and complexed forms of the enzyme (Table 2). The measurements revealed that the accessible surface area of the amino acid residues of the P_1_ subsite (His12, His 119 and Lys 41) was largely affected upon binding with the inhibitors. The observed results correlate well with the results of the docking study, suggesting that the inhibitors do bind to the P_1_ subsite of RNase A.

**Table 2:** Changes in accessible surface area (*ΔASA*) of the interacting residues between the uncomplexed and complexed forms of RNase A.

Amino Acid Residue	*ASA (*Å^2^) in RNase A	*ΔASA* (Å^2^) for Different Inhibitors			
		U-ol-Acid (8)	C-ol-Acid (18)	U-di-Acid (10)	C-di-Acid (21)
Lys 7	88.03	42.88	41.75	29.62	43.82
His 12	12.64	7.09	7.76	9.21	7.53
Arg 39	142.03	25.81	29.72	5.06	30.79
Lys 41	36.39	26.80	31.58	33.00	31.31
His 119	85.05	33.62	28.29	26.69	26.64

## 3. Experimental Section

### 3.1. General Methods

All reagents and fine chemicals were purchased from commercial suppliers and were used without further purification. Column chromatography was performed with silica gel (230–400 mesh). Solvents were dried and distilled following standard methods. TLC was carried out on precoated plates (Merck silica gel 60, f_254_). ^1^H and ^13^C-NMR for the compounds were recorded at 200 and 50 MHz, respectively, using a Bruker NMR instrument. For ^1^H and ^13^C-NMR spectra in D_2_O, CH_3_CN was used as internal standard. Chemical shifts are reported in parts per million (ppm, δ scale). Methylene carbons have been identified using DEPT spectrum. Melting points were determined in open-end capillary tubes. Bovine pancreatic RNase A, RNA (*Torula utilis*), and 2',3'-cCMP were purchased from commercial suppliers. UV-vis measurements were made using a UV-vis spectrophotometer (Shimadzu 2450). Concentrations of the solutions were estimated spectrophotometrically using the following data: ε_278.5_ = 9800 M^−1^·cm^−1^ (RNase A) [45] and ε_268_ = 8500 M^−1^·cm^−1^ (2',3'-cCMP) [22]. CD measurements were carried out on a Jasco-810 automatic recording spectrophotometer. Fluorescence measurements were carried out using a Horiba Jobin Yvon Fluoromax-4 Spectrofluorimeter.

### 3.2. General Procedure for Carboxylic Acid–Amine Coupling Reaction

To a well-stirred solution of carboxylic acid (**1**, **11)** (1 mmol) in DMF (5 mL) at 0 °C was added EDC-HCl (1.2 mmol) followed by triethylamine (1.2 mmol) and the stirring was further continued at this temperature. After 10 min, HOBT (1.2 mmol) was added and the reaction mixture was allowed to warm back to room temperature. Amine (1 mmol) was added to the resulting solution and the stirring was allowed to continue overnight. The reaction mixture was then poured into a brine solution (50 mL) and extracted with EtOAc (3 × 20 mL). Organic extracts were pooled together, dried over anhydrous Na_2_SO_4_, filtered, and the filtrate was evaporated under reduced pressure. The resulting residue was purified by column chromatography over silica gel (EtOAc/petroleum ether) to obtain pure coupling products (**4**, **6**, **9**, **12**, **15**, **17**, **19**).

### 3.3. General Procedure for Deprotection of –TBDMS Group

To a well-stirred solution of compound (**4**, **6**) (1 mmol) in methanol (10 mL), was added CH_3_COCl (cat). The resulting solution was allowed to stir for 2 h at room temperature. The solvent was evaporated under reduced pressure and the resulting residue was purified by column chromatography over silica gel (DCM/MeOH) to obtain pure products (**5**, **7**).

### 3.4. General Procedure for the Deprotection of –BOC Group/Simultaneous Deprotection of –BOC Group and –TBDMS Group

The compound (**12**, **15**, **19**) (1 mmol) was dissolved in 1:1 TFA/DCM (5 mL) and the resulting solution was stirred at room temperature for 4 h. The solvent was evaporated under reduced pressure and the resulting residue was purified by column chromatography over silica gel (DCM/MeOH) to obtain pure products (**13**, **16**, **20**). 

### 3.5. General Procedure for Ester Hydrolysis

To a well-stirred solution of the compound (**7**, **9**, **13**, **20**) (1 mmol) in methanol (5 mL) at 0 °C was added 1 N NaOH solution (2 mL) dropwise. The resulting solution was allowed to warm back up to room temperature and stirred for 1 h. After evaporation of methanol under reduced pressure, the resulting residue was dissolved in water (10 mL) and neutralized with acidic amberlyte. The resulting mixture was filtered, and the filtrate was evaporated under reduced pressure. The resulting residue was purified by column chromatography over silica gel (DCM/MeOH) to obtain pure products (**8**, **10**, **14**, **21**).

*N,N-Bis-[2-(tert-butyldimethylsilyloxy)-ethyl]-2-(2,4-dioxo-3,4-dihydro-2H-pyrimidin-1-yl)-acetamide* (**4**): Compound **1** (0.68 g, 3.99 mmol) was converted to compound **4** (1.28 g, 66%) following the general procedure in Section 3.2. R_f_ = 0.4 [30% EtOAc in pet ether]. White solid. M.P: 122–124 °C. ^1^H-NMR (200 MHz, CDCl_3_): δ 0.02 (s, 6H), 0.06 (s, 6H), 0.86 (s, 9H), 0.88 (s, 9H), 3.47 (t, *J* = 5.5 Hz, 2H), 3.59 (t, *J* = 5.1 Hz, 2H), 3.71–3.80 (m, 4H), 4.66 (s, 2H), 5.69 (d, *J* = 8.0 Hz, 1H), 7.11 (d, *J* = 7.8 Hz, 1H), 9.28 (bs, 1H). ^13^C-NMR (50 MHz, CDCl_3_): δ −5.3, −5.2, 18.3, 18.5, 26.0, 26.1, 48.1 (CH_2_), 49.2 (CH_2_), 51.1 (CH_2_), 61.0(CH_2_), 61.5 (CH_2_), 102.1, 145.4, 151.2, 163.9, 167.0. HRMS (ESI^+^): *m*/*z* calcd for C_22_H_44_N_3_O_5_Si_2_ (M+H)^+^: 486.2820; found: 486.2827.

*2-(2,4-Dioxo-3,4-dihydro-2H-pyrimidin-1-yl)-N,N-bis-(2-hydroxy-ethyl) acetamide* (**5**): Compound **5** [38] (0.39 g, 81%) was obtained from compound **4** (0.9 g, 1.85 mmol) following the general procedure in Section 3.3. White solid. R_f_ = 0.4 [15% MeOH in DCM]. ^1^H-NMR (200 MHz, DMSO-*d_6_*): δ 3.31–3.58 (m, 8H), 4.67 (s, 2H), 4.75–4.80 (m, 1H), 5.01–5.05 (m, 1H), 5.54 (d, *J* = 7.8 Hz, 1H), 7.47 (d, *J* = 7.8 Hz, 1H), 11.23 (bs, 1H).

*{[2-(tert-Butyldimethylsilyloxy) ethyl]-[2-(2,4-dioxo-3,4-dihydro-2H-pyrimidin-1-yl-acetyl] amino}acetic acid ethyl ester* (**6**): Compound **1** (0.93 g, 5.47 mmol) was converted to compound **6** (1.40 g, 62%) following the general procedure in Section 3.2. R_f_ = 0.3 [40% EtOAc in pet ether]. White solid. M.P: 171–172 °C. The compound was obtained as a mixture of rotamers as indicated by the NMR spectra. ^1^H-NMR (200 MHz, CDCl_3_): δ 0.01 (s, 6H), 0.06 (s, 6H), 0.86 (s, 9H), 0.87 (s, 9H), 1.20–1.32 (m, 3H), 3.52–3.56 (m, 2H), 3.70–3.81 (m, 2H), 4.10–4.24 (m, 4H), 4.30 (s, 2H), 4.46 (s, 2H), 4.71 (s, 2H), 5.68–5.73 (m, 1H), 7.13–7.26 (m, 1H), 9.39 (bs, 1H), 9.51 (bs, 1H). ^13^C-NMR (50 MHz, CDCl_3_): δ −5.3, 14.3, 18.4, 26.0, 26.1, 47.9 (CH_2_), 48.6 (CH_2_), 50.8 (CH_2_), 61.4 (CH_2_), 61.6 (CH_2_), 62.2 (CH_2_), 102.3, 145.4, 151.1, 163.9, 167.5, 168.9, 172.9. HRMS (ESI^+^): *m*/*z* calcd for C_18_H_32_N_3_O_6_Si (M+H)^+^: 414.2060; found: 414.2088.

*[[2-(2,4-Dioxo-3,4-dihydro-2H-pyrimidin-1-yl-acetyl]-(2-hydroxyethyl)-amino] acetic acid ethyl ester* (**7**): Compound **7** (0.48 g, 55%) was generated from compound **6** (1.21 g, 2.90 mmol) following the general procedure in Section 3.3. R_f_ = 0.4 [10% MeOH in DCM]. Light yellow gum. The compound was obtained as a mixture of rotamers as indicated by the NMR spectra. ^1^H-NMR (200 MHz, DMSO-*d_6_*): δ 1.14–1.26 (m, 3H), 3.34–3.58 (m, 4H), 4.01–4.18 (m, 4H), 4.34 (s, 2H), 4.53 (s, 2H), 4.74 (s, 2H), 5.53–5.57 (m, 1H), 7.34–7.45 (m, 1H), 11.25 (bs, 1H). ^13^C-NMR (50 MHz, DMSO-*d_6_*): δ 14.0, 48.0 (CH_2_), 49.9 (CH_2_), 50.7 (CH_2_), 58.3 (CH_2_), 58.9 (CH_2_), 60.5 (CH_2_), 61.03(CH_2_), 100.6, 146.5, 151.0, 163.9, 167.4, 167.5, 169.1, 169.4. HRMS (ESI^+^): *m*/*z* calcd for C_12_H_17_N_3_O_6_Na (M+Na)^+^: 322.1015; found: 322.1003.

*[[2-(2,4-Dioxo-3,4-dihydro-2H-pyrimidin-1-yl-acetyl]-(2-hydroxyethyl) amino] acetic acid* (**8**): Compound **7** (0.4 g, 1.34 mmol) was converted to compound **8** (0.22 g, 62%) following the general procedure in Section 3.5. R_f_ = 0.2 [20% MeOH in DCM]. White solid. M.P: 177–179 °C. The compound was obtained as a mixture of rotamers as indicated by the NMR spectra. ^1^H-NMR (200 MHz, D_2_O): δ 3.54–3.82 (m, 4H), 4.19 (s, 2H), 4.37 (s, 2H), 4.57 (s, 2H), 4.90 (s, 2H), 5.80–5.87 (m, 1H), 7.50–7.56 (m, 1H). ^13^C-NMR (50 MHz, D_2_O): δ 49.2 (CH_2_), 50.0 (CH_2_), 50.6 (CH_2_), 51.0 (CH_2_), 59.3 (CH_2_), 59.4 (CH_2_), 102.2, 148.1, 152.6, 167.2, 170.0, 173.3. HRMS (ESI^+^): *m*/*z* calcd for C_10_H_13_N_3_O_6_Na (M+Na)^+^: 294.0702; found: 294.0707.

*{[2-(2,4-Dioxo-3,4-dihydro-2H-pyrimidin-1-yl-acetyl] ethoxycarbonylmethylamino}acetic acid ethyl ester* (**9**): Compound **1** (0.52 g, 3.05 mmol) was transformed to compound **9** (0.51 g, 49%) following the general procedure in Section 3.2. R_f_ = 0.5 [70% EtOAc in pet ether]. White solid. M.P: 140–142 °C. ^1^H-NMR (200 MHz, CDCl_3_): δ 1.22–1.34 (m, 6H), 4.12–4.31 (m, 8H), 4.61 (s, 2H), 5.73 (d, *J* = 8.0 Hz, 1H), 7.20 (d, *J* = 7.8 Hz, 1H), 9.09 (bs, 1H). ^13^C-NMR (50 MHz, CDCl_3_): δ 14.3, 47.7 (CH_2_), 49.0 (CH_2_), 50.2 (CH_2_), 61.8 (CH_2_), 62.5 (CH_2_), 102.6, 145.2, 151.0, 163.7, 167.6, 168.6. HRMS (ESI^+^): *m*/*z* calcd for C_14_H_19_N_3_O_7_Na (M+Na)^+^: 364.1121; found: 364.1109.

*{*Carboxymethyl*-[2-(2,4-dioxo-3,4-dihydro-2H-pyrimidin-1-yl) acetyl] amino}acetic acid* (**10**): Compound **9** (0.42 g, 1.23 mmol) was converted to compound **10** (0.26 g, 74%) following the general procedure in Section 3.5. R_f_ = 0.2 [30% MeOH in DCM]. White solid. M.P: 118–120 °C ^1^H-NMR (200 MHz, DMSO-*d_6_*): δ 4.00 (s, 2H), 4.25 (s, 2H), 4.62 (s, 2H), 5.55 (d, *J* = 7.2 Hz, 1H), 7.43 (d, *J* = 7.6 Hz, 1H), 11.28 (s, 1H). ^13^C-NMR (50 MHz, DMSO-*d_6_*): δ 47.7 (CH_2_), 48.4 (CH_2_), 49.2 (CH_2_), 100.7, 146.4, 151.0, 163.8, 167.7, 170.3. HRMS (ESI^+^): *m*/*z* calcd for C_10_H_11_N_3_O_7_Na (M+Na)^+^: 308.0495; found: 308.0475.

*{[4-[Bis(1,1-dimethylethoxy)carbonyl]amino-2-oxo-2H-pyrimidin-1-yl] acetylamino}acetic acid ethyl ester (**12**)*: Compound **11** (0.39 g, 1.05 mmol) was converted to compound **12** (0.30 g, 62%) following the general procedure in Section 3.2. R_f_ = 0.5 [60% EtOAc in pet ether]. White solid. M.P: 118–120 °C. ^1^H-NMR (200 MHz, CDCl_3_): δ 1.18–1.25 (m, 3H), 1.52 (s, 18H), 3.95 (d, *J* = 5.6 Hz, 2H), 4.12 (q, *J* = 7.2 Hz, 2H), 4.58 (s, 2H), 7.08 (d, *J* = 7.2 Hz, 1H), 7.69–7.76 (m, 1H). ^13^C-NMR (50 MHz, CDCl_3_): δ 14.1, 27.7, 41.4 (CH_2_), 52.8 (CH_2_), 61.4 (CH_2_), 85.0, 96.7, 149.0, 149.4, 152.6, 155.5, 162.7, 167.1, 169.4. HRMS (ESI^+^): *m*/*z* calcd for C_20_H_30_N_4_O_8_Na (M+Na)^+^: 477.1961; found: 477.1975.

*[2-(4-Amino-2-oxo-2H-pyrimidin-1-yl) acetylamino] acetic acid ethyl ester* (**13**): Compound **12** (0.28 g, 0.62 mmol) was converted to compound **13** (0.11 g, 72%) following the general procedure in Section 3.4. R_f_ = 0.3 [5% MeOH in DCM]. White solid. M.P: 155–158 °C. ^1^H-NMR (200 MHz, DMSO-*d_6_*): δ 1.17 (t, *J* = 7.2 Hz, 3H), 3.86 (d, *J* = 5.8 Hz, 2H), 4.07 (q, *J* = 7.2 Hz, 2H), 4.42 (s, 2H), 5.82 (d, *J* = 7.2 Hz, 1H), 7.65 (d, *J* = 7.4 Hz, 1H), 7.90 (bs, 1H), 8.09 (bs, 1H), 8.61 (t, *J* = 5.8 Hz, 1H). ^13^C-NMR (50 MHz, DMSO-*d_6_*): δ 14.5, 41.2 (CH_2_), 50.9 (CH_2_), 61.0 (CH_2_), 93.8, 148.8, 153.5, 164.4, 168.0, 170.0. HRMS (ESI^+^): *m*/*z* calcd for C_10_H_15_N_4_O_4_ (M+H)^+^: 255.1093; found: 255.1076.

*[2-(4-Amino-2-oxo-2H-pyrimidin-1-yl) acetylamino] acetic acid* (**14**): Compound **13** (0.09 g, 0.35 mmol) was transformed to compound **14** (0.05 g, 67%) following the general procedure in Section 3.5. R_f_ = 0.3 [20% *MeOH* in DCM]. Yellowish white solid. M.P: > 200 °C. ^1^H-NMR (200 MHz, DMSO-*d_6_*): δ 3.61–3.95 (m, 2H), 4.54 (s, 2H), 6.22 (d, *J* = 7.2 Hz, 1H), 7.99 (d, *J* = 7.4 Hz, 1H), 8.77 (s, 1H), 8.94 (s, 1H), 10.17 (s, 1H). ^13^C-NMR (50 MHz, DMSO-*d_6_*): δ 40.8 (CH_2_), 50.4 (CH_2_), 93.1, 147.4, 150.6, 160.1, 166.4, 170.8. HRMS (ESI^+^): *m*/*z* calcd for C_8_H_11_N_4_O_4_ (M+H)^+^: 227.0780; found: 227.0784.

*[4-[Bis(1,1-dimethylethoxy)carbonyl]amino-2-oxo-2H-pyrimidin-1-yl]-N,N-bis-[2(tertbutyldimethyl silyloxy)ethyl]acetamide* (**15**): Compound **15** (0.55 g, 69%) was obtained from compound **11** (0.43 g, 1.16 mmol) following the general procedure in Section 3.2. R_f_ = 0.3 [25% EtOAc in pet ether]. Colourless gum. ^1^H-NMR (200 MHz, CDCl_3_): δ −0.03 (s, 6H), 0.01 (s, 6H), 0.81 (s, 9H), 0.83 (s, 9H), 1.49 (s, 18H), 3.43 (t, *J* = 5.6 Hz, 2H), 3.64–3.74 (m, 6H), 4.70 (s, 2H), 7.00 (d, *J* = 7.4 Hz, 1H), 7.52 (d, *J* = 7.4 Hz, 1H). ^13^C-NMR (50 MHz, CDCl_3_): δ −5.4, 18.2, 18.3, 26.0, 27.7, 49.3 (CH_2_), 49.5 (CH_2_), 51.1 (CH_2_), 61.0 (CH_2_), 61.2 (CH_2_), 84.8, 96.1, 149.1, 149.6, 155.1, 162.6, 167.1. HRMS (ESI^+^): *m*/*z* calcd for C_32_H_61_N_4_O_8_Si_2_ (M+H)^+^: 685.4028; found: 685.4038.

*2-(4-Amino-2-oxo-2H-pyrimidin-1-yl)-N,N-bis-(2-hydroxyethyl) acetamide* (**16**): Compound **15** (0.48 g, 0.70 mmol) was transformed to compound **16** (0.13 g, 71%) following the general procedure in Section 3.4. R_f_ = 0.3 [15% MeOH in DCM]. White solid. M.P: 150 °C (decomposed). ^1^H-NMR (200 MHz, DMSO-*d_6_*): δ 2.99–3.04 (m, 1H), 3.34–3.68 (m, 8H), 4.65 (s, 2H), 5.78 (d, *J* = 7.2 Hz, 1H), 7.51 (d, *J* = 7.2 Hz, 1H), 7.75 (bs, 2H). ^13^C-NMR (50 MHz, DMSO-*d_6_*): δ 48.8 (CH_2_), 49.3 (CH_2_), 49.9 (CH_2_), 58.7 (CH_2_), 59.0 (CH_2_), 93.2, 148.1, 154.4, 164.8, 167.3. HRMS (ESI^+^): *m*/*z* calcd for C_10_H_17_N_4_O_4_ (M+H)^+^: 257.1250; found: 257.1255.

*{[4-[Bis(1,1-dimethylethoxy)carbonyl]amino-2-oxo-2H-pyrimidin-1-yl-acetyl]-[2-(tert-butyldimethylsilyloxy) ethyl] acetic acid ethyl ester* (**17**): Compound **11** (0.46 g, 1.24 mmol) was converted to compound **17** (0.52 g, 69%) following the general procedure in Section 3.2. R_f_ = 0.4 [40% EtOAc in pet ether]. Colorless gum. The compound was obtained as a mixture of rotamers as indicated by the NMR spectra. ^1^H-NMR (200 MHz, CDCl_3_): δ −0.03–0.01 (m, 6H), 0.81 (s, 9H), 0.83 (s, 9H), 1.15–1.27 (m, 3H), 1.49 (s, 18H), 3.33–3.80 (m, 4H), 4.05–4.18 (m, 4H), 4.40 (s, 2H), 4.49 (s, 2H), 4.75 (s, 2H), 6.97–7.03 (m, 1H), 7.54–7.62 (m, 1H). ^13^C-NMR (50 MHz, CDCl_3_): δ −5.4, 14.2, 18.2, 25.9, 27.7, 49.0 (CH_2_), 49.3 (CH_2_), 51.1 (CH_2_), 51.2 (CH_2_), 61.2 (CH_2_), 61.6 (CH_2_), 61.8 (CH_2_), 62.0 (CH_2_), 84.8, 96.2, 149.0, 149.1, 149.5, 155.0, 162.7, 162.8, 167.4, 167.5, 168.8, 169.7. HRMS (ESI^+^): *m*/*z* calcd for C_28_H_49_N_4_O_9_Si (M+H)^+^: 613.3269; found: 613.3278.

*[[2-(4-Amino-2-oxo-2H-pyrimidin-1-yl) acetyl]-(2-hydroxyethyl) amino] acetic acid* (**18**): Compound **17** (0.40 g, 0.65 mmol) was hydrolyzed to corresponding acid following the general procedure in Section 3.5. The *crude* residue obtained was subjected to TFA treatment as mentioned in the general procedure in Section 3.4, and purified by column chromatography over silica gel to obtain **18** (0.10 g, 58%). R_f_ = 0.2 [30% MeOH in DCM]. White solid. M.P: 184–188 °C. The compound was obtained as a mixture of rotamers, as indicated by the NMR spectra. ^1^H-NMR (200 MHz, DMSO-*d_6_*): δ 3.35–3.59 (m, 4H), 4.01 (s, 2H), 4.24 (s, 2H), 4.54 (s, 2H) 4.75 (s, 2H), 5.84 (d, *J* = 7.0 Hz, 1H), 7.55 (d, *J* = 7.0 Hz, 1H), 7.97 (bs, 2H). ^13^C-NMR (50 MHz, DMSO-*d_6_*): δ 47.9 (CH_2_), 49.1 (CH_2_), 49.9 (CH_2_), 58.9 (CH_2_), 93.4, 148.6, 152.8, 163.6, 167.6, 170.7, 171.1. HRMS (ESI^+^): *m*/*z* calcd for C_10_H_15_N_4_O_5_ (M+H)^+^: 271.1042; found: 271.1039.

*{[4-[Bis(1,1-dimethylethoxy)carbonyl]amino-2-oxo-2H-pyrimidin-2-yl-acetyl] ethoxycarbonylmethylamino}acetic acid ethyl ester* (**19**): Compound **19** (0.29 g, 54%) was generated from compound **11** (0.37 g, 1.00 mmol) following the general procedure in Section 3.2. R_f_ = 0.4 [50% EtOAc in pet ether]. Colorless gum. ^1^H-NMR (200 MHz, CDCl_3_): δ 1.17–1.30 (m, 6H), 1.51 (s, 18H), 4.07–4.25 (m, 6H), 4.32 (s, 2H), 4.67 (s, 2H), 7.05 (d, *J* = 7.4 Hz, 1H), 7.62 (d, *J* = 7.4 Hz, 1H). ^13^C-NMR (50 MHz, CDCl_3_): δ 14.2, 27.8, 49.1 (CH_2_), 49.4 (CH_2_), 50.5 (CH_2_), 61.6 (CH_2_), 62.1 (CH_2_), 85.0, 96.5, 149.0, 149.5, 155.1, 162.8, 167.9, 168.7, 168.9. HRMS (ESI^+^): *m*/*z* calcd for C_24_H_37_N_4_O_10_ (M+H)^+^: 541.2510; found: 541.2516.

*{[2-(4-Amino-2-oxo-2H-pyrimidin-1-yl) acetyl] ethoxycarbonylmethylamino}acetic acid ethyl ester* (**20**): Compound **19** (0.27 g, 0.50 mmol) was converted to compound **20** (0.09 g, 56%) following the general procedure in Section 3.4. R_f_ = 0.3 [3% MeOH in DCM]. White solid. M.P: 152–154 °C. ^1^H-NMR (200 MHz, DMSO-*d_6_*): δ 1.14–1.27 (m, 6H), 4.00–4.22 (m, 6H), 4.39 (s, 2H), 4.80 (s, 2H), 6.10 (d, *J* = 7.6 Hz, 1H), 7.81 (d, *J* = 7.6 Hz, 1H), 9.52 (bs, 1H), 9.67 (bs, 1H). ^13^C-NMR (50 MHz, DMSO-*d_6_*): δ 14.0, 48.5 (CH_2_), 49.1 (CH_2_), 60.6 (CH_2_), 61.1 (CH_2_), 93.5, 148.2, 150.2, 160.6, 167.3, 168.5, 168.8. HRMS (ESI^+^): *m*/*z* calcd for C_14_H_21_N_4_O_6_ (M+H)^+^: 341.1461; found: 341.1452.

*{[2-(4-Amino-2-oxo-2H-pyrimidin-1-yl)-acetyl] carboxymethylamino}acetic acid* (**21**): Compound **20** (0.07 g, 0.20 mmol) was transformed to compound **21** (0.04 g, 66%) following the general procedure in Section 3.5. R_f_ = 0.15 [40% MeOH in DCM]. Eluent: 30%–50% MeOH in DCM. White solid. M.P: 190–193 °C. ^1^H-NMR (200 MHz, D_2_O): δ 4.23 (s, 2H), 4.39 (s, 2H), 4.89 (s, 2H), 6.24 (d, *J* = 7.4 Hz, 1H), 7.79 (d, *J* = 7.8 Hz, 1H). ^13^C-NMR (50 MHz, DMSO-*d_6_*): δ 48.8 (CH_2_), 49.3 (CH_2_), 49.9 (CH_2_), 93.4, 148.4, 152.2, 163.5, 167.8, 170.4, 170.7. HRMS (ESI^+^): *m*/*z* calcd for C_10_H_13_N_4_O_6_ (M+H)^+^: 285.0835; found: 285.0852.

### 3.6. Comparative Agarose Gel-Based Assay

Inhibition of RNase A was assayed qualitatively by the degradation of RNA in an agarose gel. In this method, 20 μL of RNase A (1 μM) was mixed with 20 μL (0.5 mM) of compounds **U-ol-ol** (**5**), **C-ol-ol** (**16**), **U-acid** (**3**), **C-acid** (**14**), **U-ol-acid** (**8**), **C-ol-acid** (**18**), **U-di-acid** (**10**), and **C-di-acid** (**21**) separately to a final volume of 50 μL and the resulting solutions incubated for 3 h. Twenty-microliter aliquots of the incubated mixtures were then mixed with 20 μL of RNA solution (10.0 mg/mL RNA, freshly dissolved in RNase free water) and incubated for another 30 min. Then 10 μL of sample buffer (containing 10% glycerol and 0.025% bromophenol blue) were added to this mixture and 15 μL from each solution were extracted and loaded onto a 1.1% agarose gel. The gel was run using a 0.04-M Tris-Acetic acid-EDTA (TAE) buffer (pH 8.0). The residual RNA was visualized by ethidium bromide staining under UV light.

### 3.7. Inhibition Kinetics with RNase A

A quantitative account of RNase A inhibition by the individual inhibitors was obtained by a UV spectroscopic method described by Anderson and co-workers [22]. The assay was performed in a 0.1-M Mes-NaOH buffer, pH 6.0 containing 0.1 M NaCl using 2',3'-cCMP as the substrate. The inhibition constants were calculated from initial velocity data using a Lineweaver–Burk plot. The slopes from the Lineweaver–Burk double reciprocal plot were plotted against the corresponding inhibitor concentrations to get inhibition constants (*K_i_*).

### 3.8. Circular Dichroism Measurements

Circular Dichroism (CD) was performed in order to monitor the changes in the secondary structure of the enzyme as a result of interaction with the inhibitors. In this method, 200 µL of RNase A (30 µM) were mixed separately with 200 µL of **U-di-acid** (**10**) (30 µM) and **C-di-acid** (**21**) (30 µM) and incubated for 3 h. From the resulting solutions, an aliquot of 300 µL was used for CD measurements, taking a 1 mm path length quartz cell. The spectra were recorded in the range of 190–240 nm with a scan rate of 50 nm/min. Three scans were accumulated for each spectrum. The secondary structure was determined using an online server, DICHROWEB [46].

### 3.9. Fluorescence Spectroscopy

Fluorescence quenching study was performed to garner an idea of the binding affinities of **U-di-acid** (**10**) and **C-di-acid** (**21**) towards RNase A. The emission spectra were recorded from 290 to 400 nm with excitation at 275 nm [47] using a 5 nm slit width. The interaction between the ligands and RNase A was investigated by titration of 3 mL solution of RNase A with successive addition of the respective ligands (0–15 μM) in a 20-mM phosphate buffer of pH 7.0. Binding constants (*K_b_*) were calculated using double-logarithm plot [48].

### 3.10. FlexX Docking

The crystal structure of RNase A (PDB entry 1FS3) was downloaded from the Protein Data Bank [49]. The 3D structures of the inhibitors were generated in *Sybyl6.92* (Tripos Inc., St. Louis, MO, USA). Minimum energy conformations were obtained with the help of the MMFF94 force field using MMFF94 charges with a gradient of 0.005 kcal/mole by 1000 iterations with all other default parameters. The ligands were docked with the protein using *FlexX* software. The ranking of the generated solutions was performed using a scoring function that estimates the free binding energy (*ΔG*) of the protein-ligand complex considering various types of molecular interactions [50]. Docked conformations were visualized using *PyMol* [51].

### 3.11. Accessible Surface Area Calculations

Accessible surface area of uncomplexed RNase A and its docked complexes were calculated using the program *NACCESS*. The structures obtained from the *FlexX* analysis were used for the calculation. The change in ASA for a particular residue X was calculated using: *ΔASA^X^* = *ASA^X^_RNase A_* − *ASA^X^_RNase A + inhibitor_*.

## 4. Conclusions

Strategically designed carboxylated acyclonucleosides have been established as moderate RNase A inhibitors. The experimental outcome points towards the possible contribution of a sugar ring in RNase A inhibition. Cytosine analogues have been proven to have better inhibitory properties than the corresponding uracil derivatives. **C-di-acid** (**21**) emerged as the most potent inhibitor of the series, having an inhibition constant (*K_i_*) value of 235 μM. It was observed that an increment in the number of carboxylic acid groups resulted in better inhibitory properties. However, the absence of the rigid ribose ring in the molecules significantly affects the inhibition properties of the synthetic nucleosides. These findings should act as a guideline for future design of inhibitors for RNase A and other members of the ribonuclease superfamily. 

## Supplementary Materials

Supplementary materials can be accessed at: http://www.mdpi.com/1420-3049/20/04/5924/s1.
