# Supplementary material for: Carboxylated Acyclonucleosides: Synthesis and RNase A Inhibition
Source: Molecules. 2015 Apr 3;20(4):5924–41. doi: 10.3390/molecules20045924 (PMC6272279; doi:10.3390/molecules20045924)
Supplement: Supplementary file 1 [file molecules-20-05924-s001.pdf]

Supplementary Materials

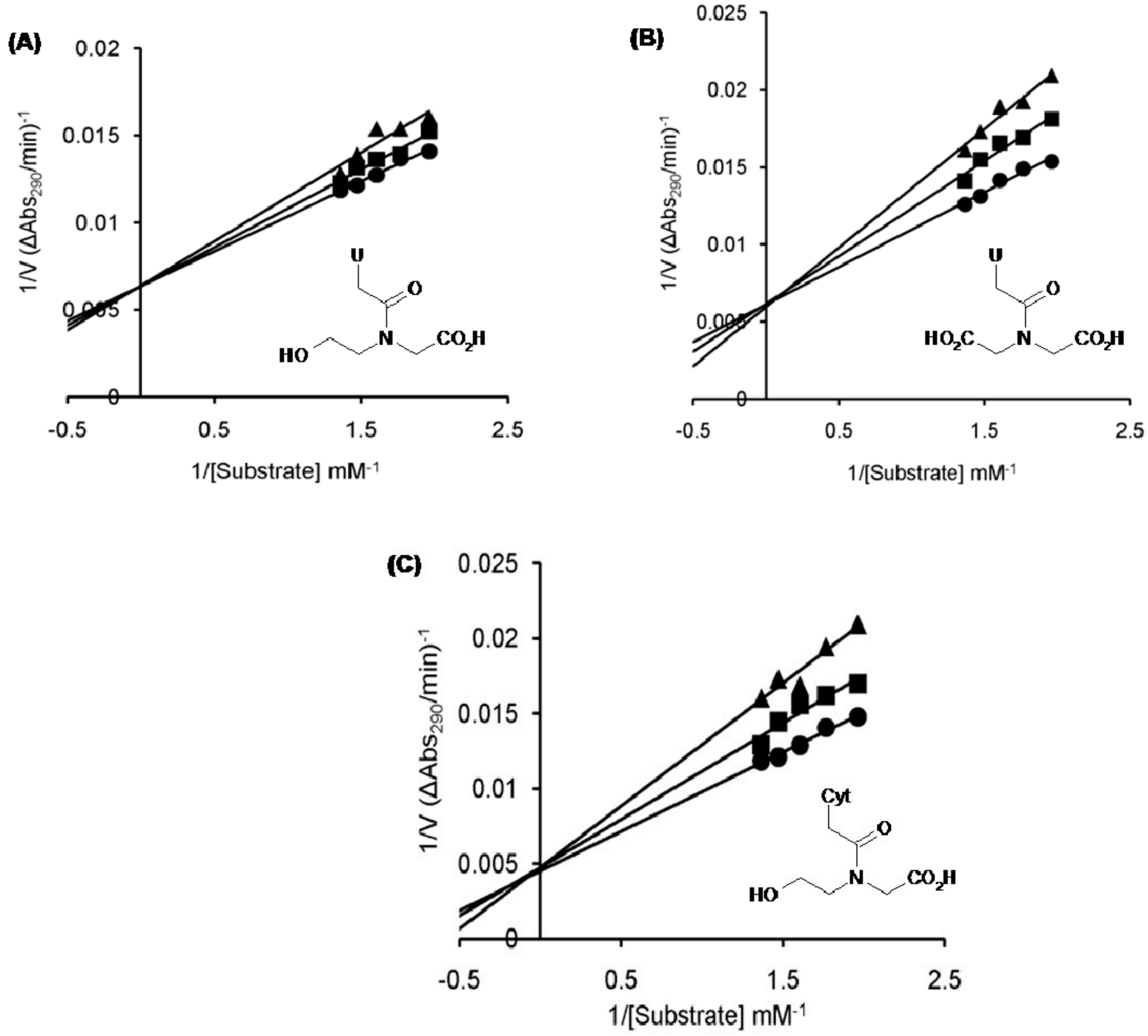

Figure S1. Lineweaver–Burk plot for inhibition of RNase A by (**A**) **U-ol-acid** (**8**) of 0.12 (▲), 0.04 (■), or 0 (●) mM, with 2′,3′-cCMP concentrations of 0.73–0.51 mM and RNase A concentration of 9.8 μM. (**B**) **U-di-acid** (**10**) of 0.165 (▲), 0.055 (■), or 0 (●) mM, with 2′,3′-cCMP concentrations of 0.73–0.51 mM and RNase A concentration of 10.2 μM. (**C**) **C-ol-acid** (**18**) of 0.20 (▲), 0.10 (■), or 0 (●) mM, with 2′,3′-cCMP concentrations of 0.72–0.50 mM and RNase A concentration of 9.9 μM.

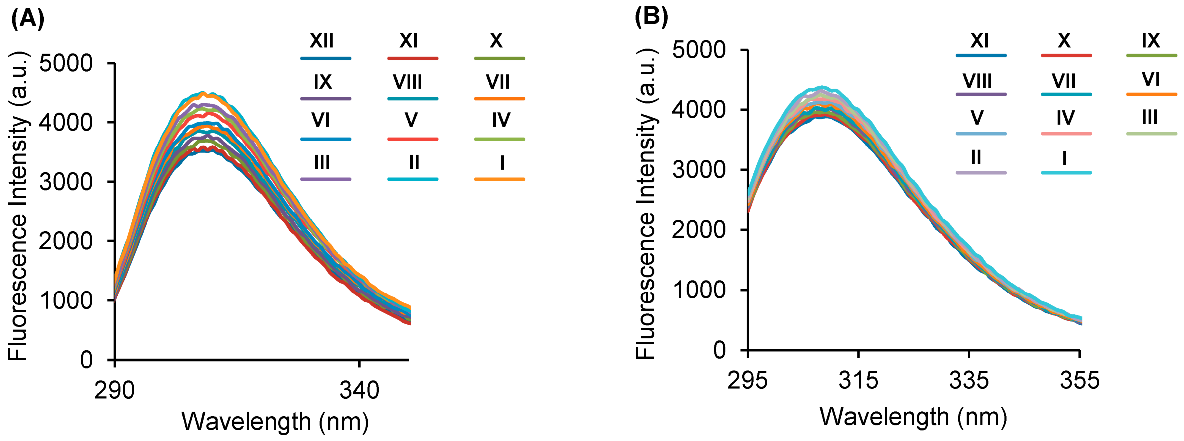

Figure S2. Fluorescence quenching spectra of RNase A in presence of (**A**) **C-di-acid** (**21**) and (**B**) **U-di-acid** (**10**). The increasing order of concentrations of the inhibitors (in µM) for each curve is as follows: (**A**) I = 0, II = 0.26, III = 0.39, IV = 0.53, V = 0.65, VI = 0.78, VII = 0.91, VIII = 1.04, IX = 1.16, X = 1.29, XI = 1.41, XII = 1.54; (**B**) I = 0, II = 0.26, III = 0.39, IV = 0.53, V = 0.65, VI = 0.78, VII = 0.91, VIII = 1.04, IX = 1.16, X = 1.29, XI = 1.41.

Table S1. Binding constants (K_b) and the number of binding sites (n) for the interactions of **U-di-acid** (**10**) and **C-di-acid** (**21**) with RNase A.

**Inhibitor**	K_b **(M^{-1})**	**No. of Binding Sites (*n*)**
**U-di-acid** (**10**)	$7.64 \pm 0.1 \times 10^4$	0.959
**C-di-acid** (**21**)	$1.44 \pm 0.02 \times 10^5$	1.257

Table S2. Distances of polar contacts between inhibitors and amino acid residues of RNase A (1FS3).

**1FS3**	**U-ol-Acid (8)**	**C-ol-Acid (18)**	**U-di-Acid (10)**	**C-di-Acid (21)**
Arg39 NH2	2.72 Å [O of C4 C=O of nucleobase]			3.2 Å [N3 of nucleobase]
Arg39 NH1	2.91 Å [O of C4 C=O of nucleobase]			3.3 Å [O of C2 C=O of nucleobase]
Lys41 CA				3.0 Å [N3 of nucleobase]
Lys41 Nζ		2.4 Å [N3 of nucleobase]	3.1 Å [C=O of amide]	
Lys7 Nζ	2.17 Å [OH of CH_2OH]	2.6 Å [OH of acid]		2.8 Å [C=O of amide]
His12 Nε2	3.21 Å [C=O of acid]	3.0 Å [C=O of amide]<br>2.1 Å [OH of CH_2OH]	2.6 Å [C=O of acid]	3.1 Å [C=O of acid]
Gln11 Nε2	2.58 Å [C=O of amide]	2.8 Å [O of C2 C=O of nucleobase]<br>2.9 Å [OH of acid]<br>2.2 Å [OH of CH_2OH]	2.9 Å [C=O of acid]	2.6 Å [C=O of acid]
Gln11 Oε1			2.2 Å [OH of acid]	
His119 Nδ1	2.57 Å [OH of acid]		1.9 Å [OH of acid]	1.9 Å [OH of acid]
Phe120 CA				3.2 Å [C=O of acid]
Phe120 N	2.83 Å [C=O of acid]	3.2 Å [C=O of amide]	2.8 Å [C=O of acid]	
Val43 O		2.0 Å [NH_2 of nucleobase]	1.9 Å [N3 of nucleobase]	

NMR Spectra

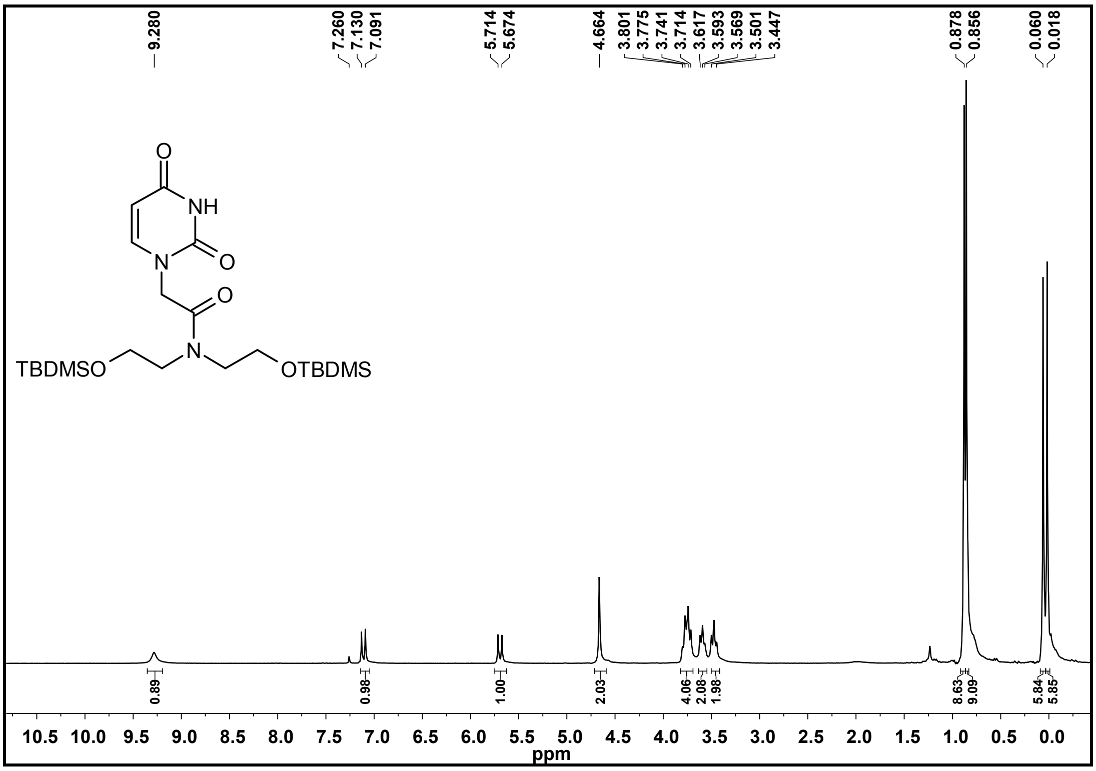

^{1}H-NMR of compound **4**.

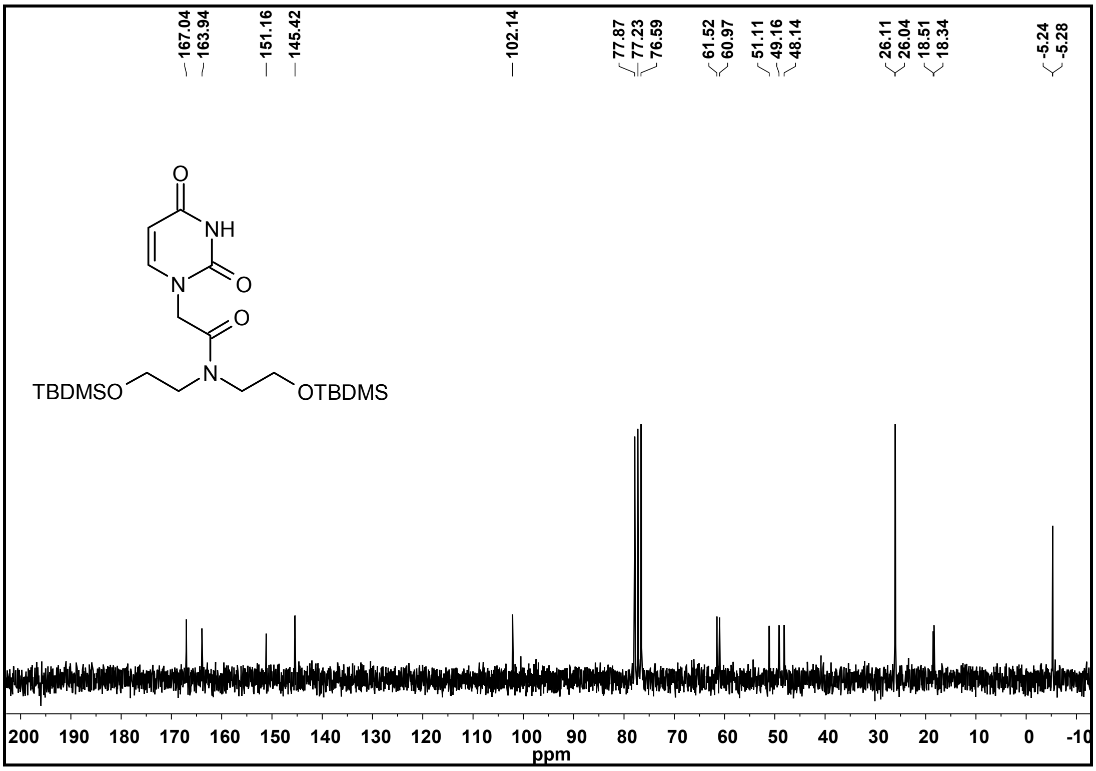

^{13}C-NMR of compound **4**.

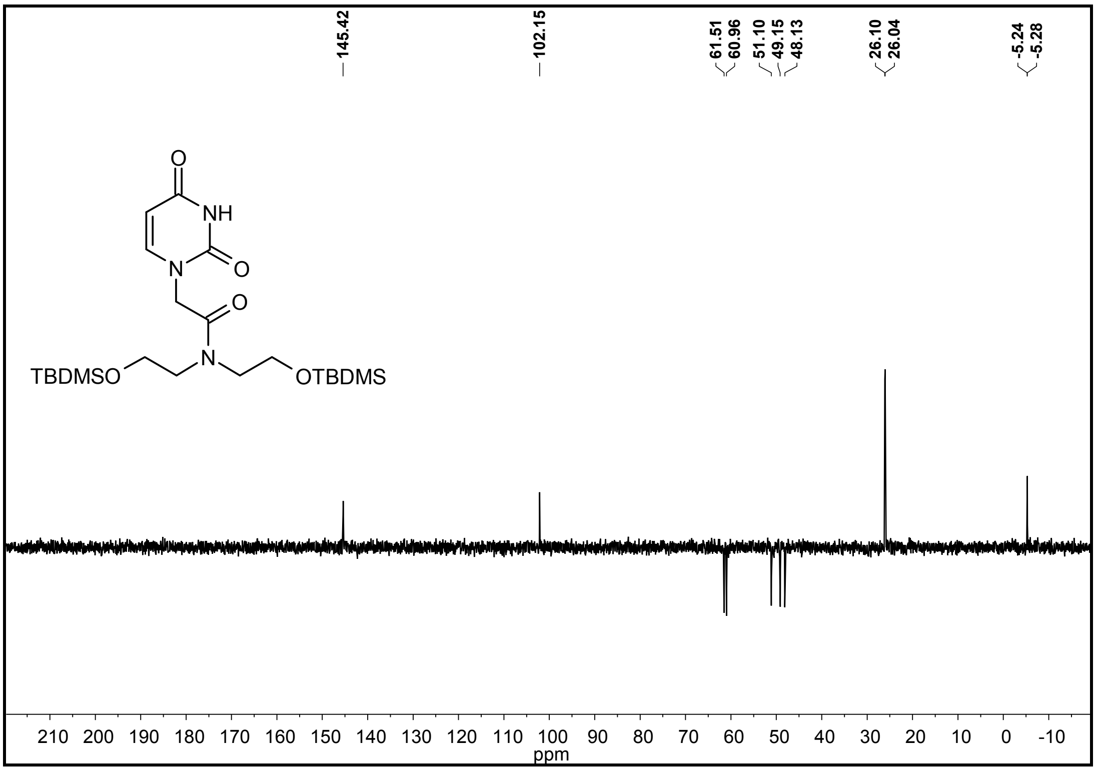

DEPT 135 NMR of compound **4**.

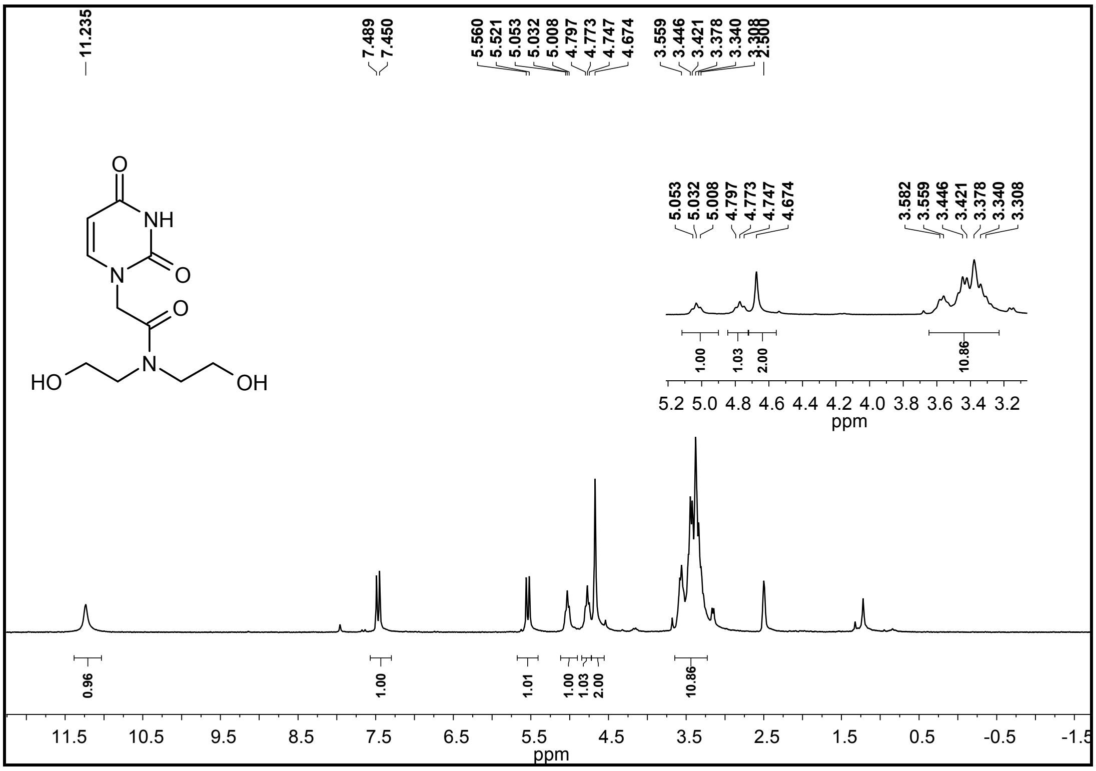

^{1}H-NMR of compound **5**.

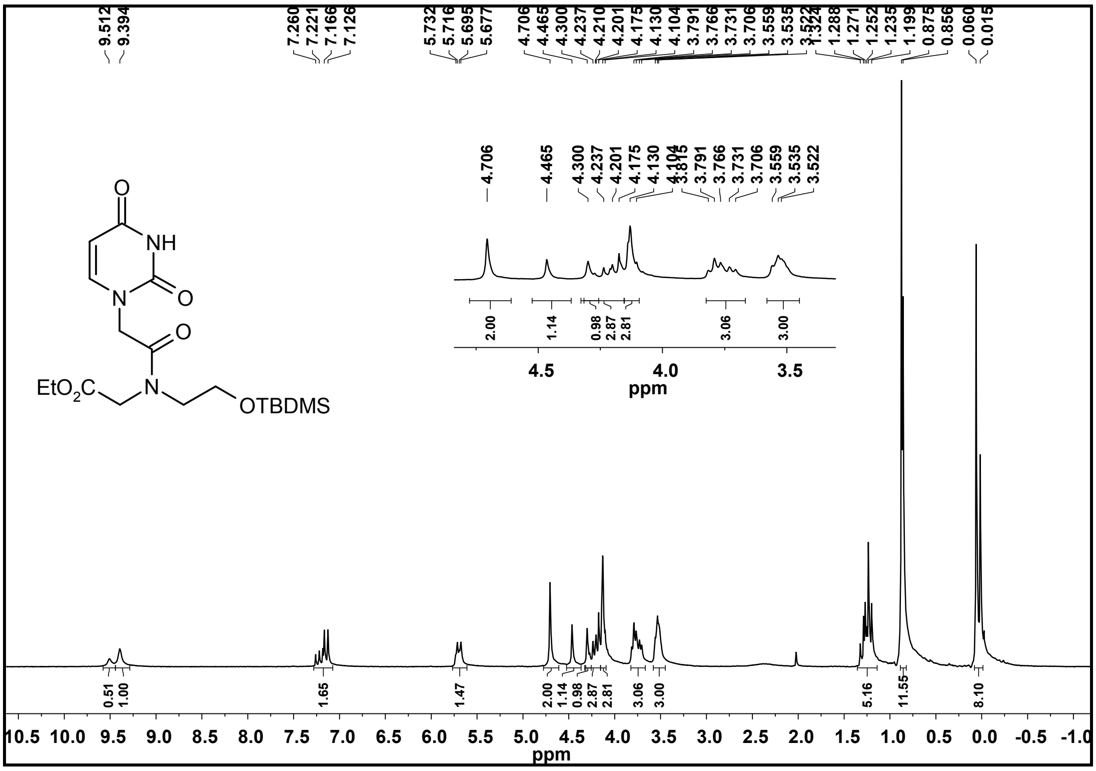

^{1}H-NMR of compound **6**.

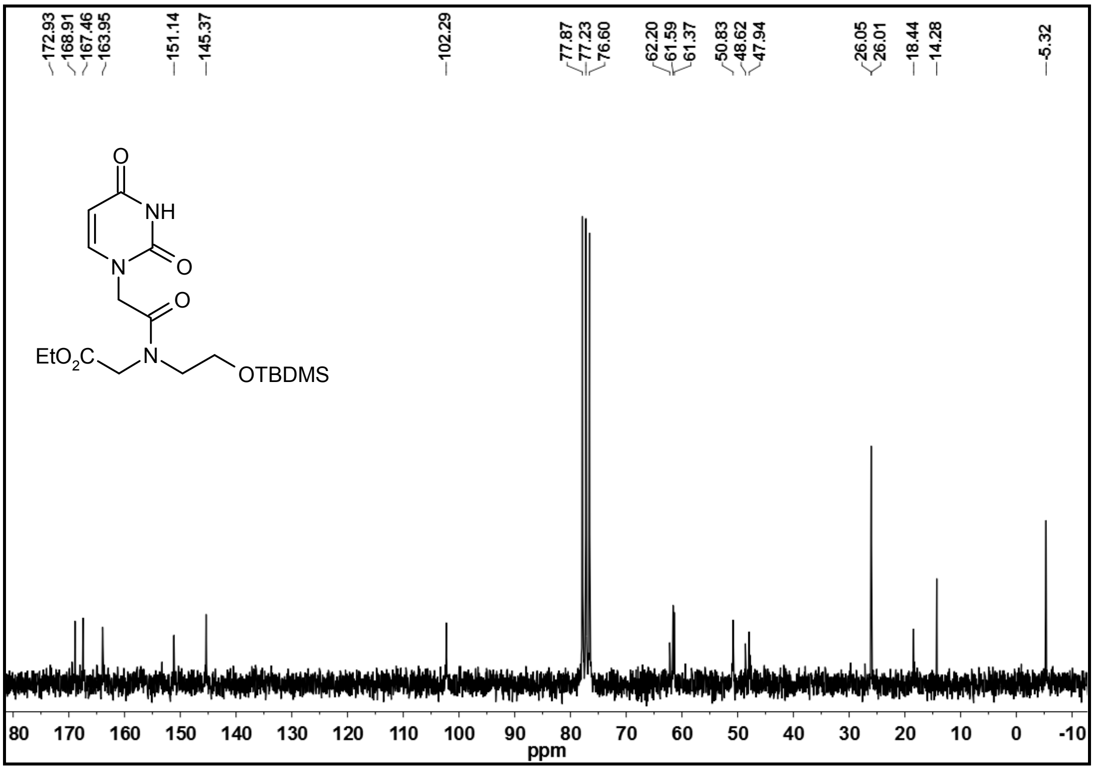

^{13}C-NMR of compound **6**.

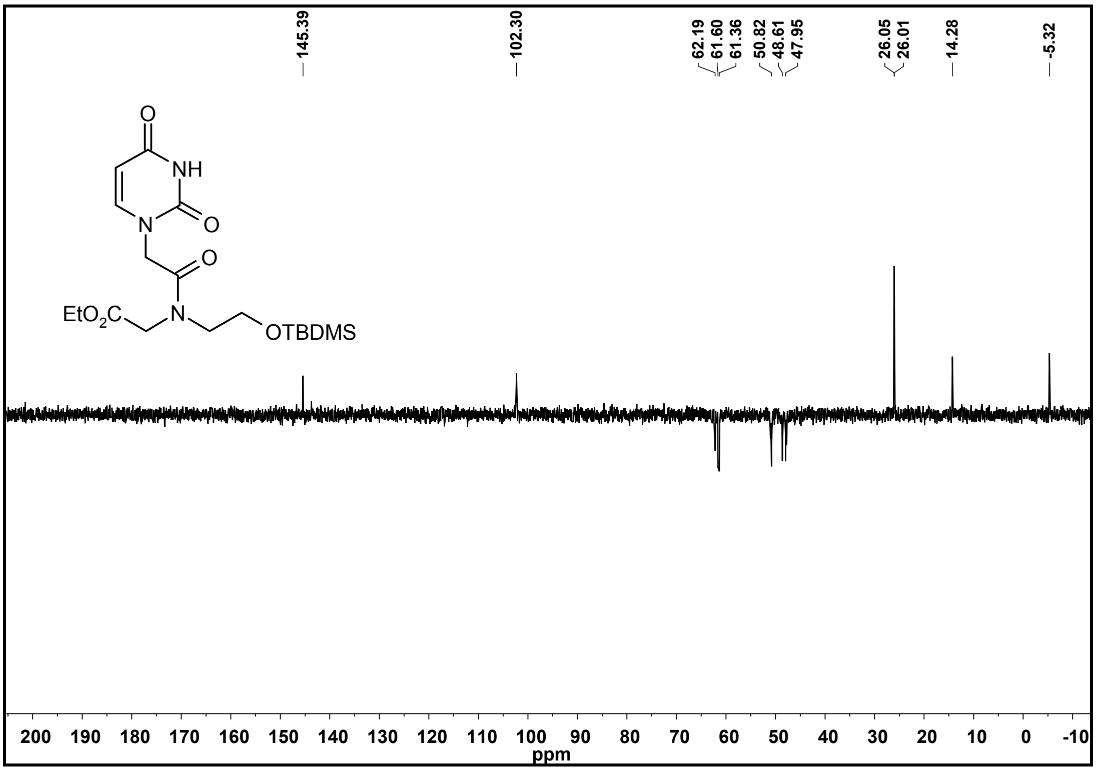

DEPT 135 NMR of compound **6**.

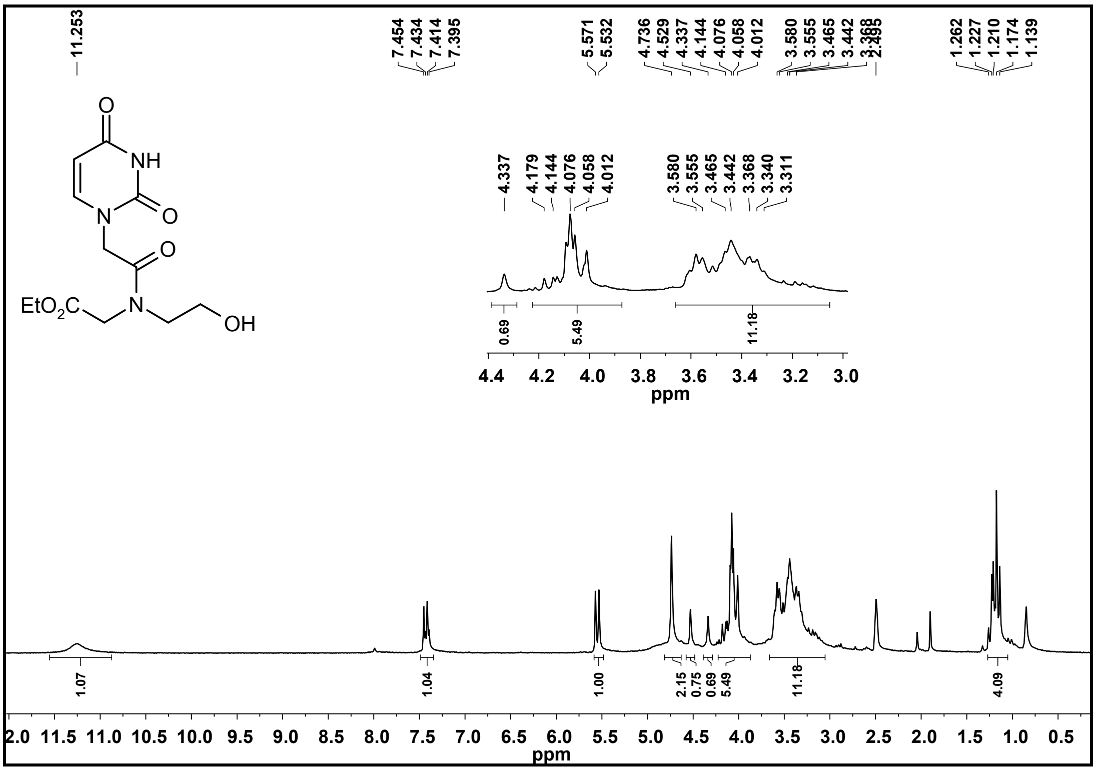

^{1}H-NMR of compound **7**.

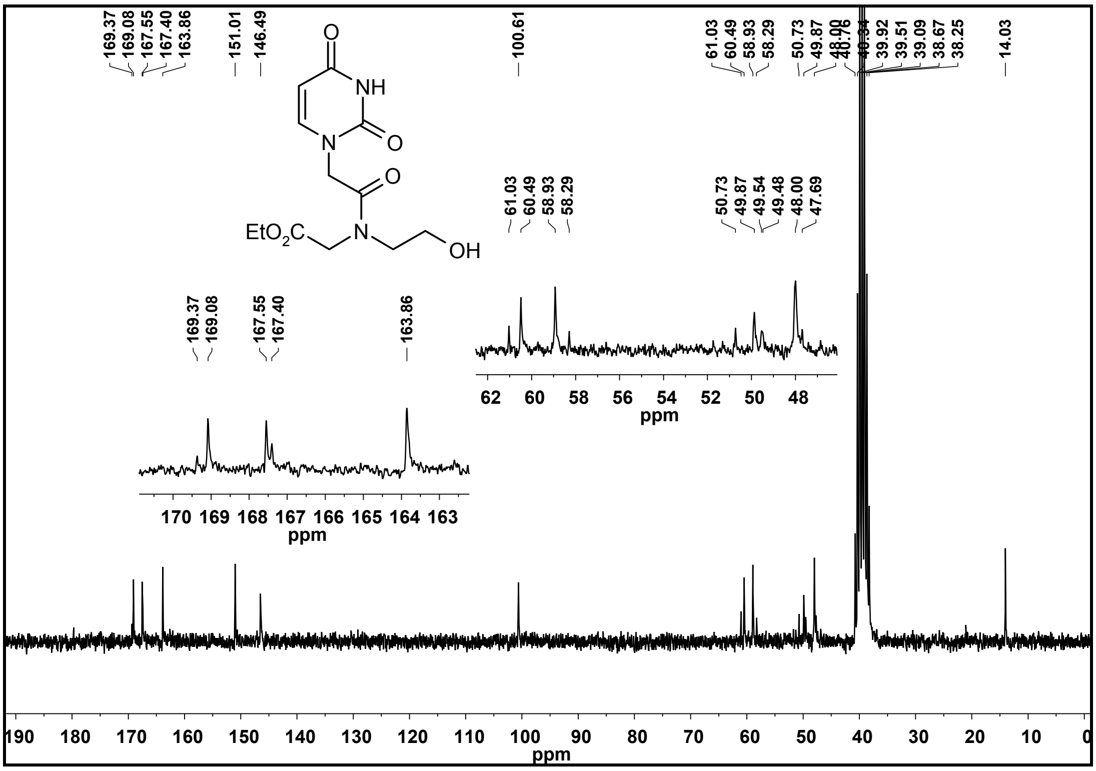

^{13}C-NMR of compound **7**.

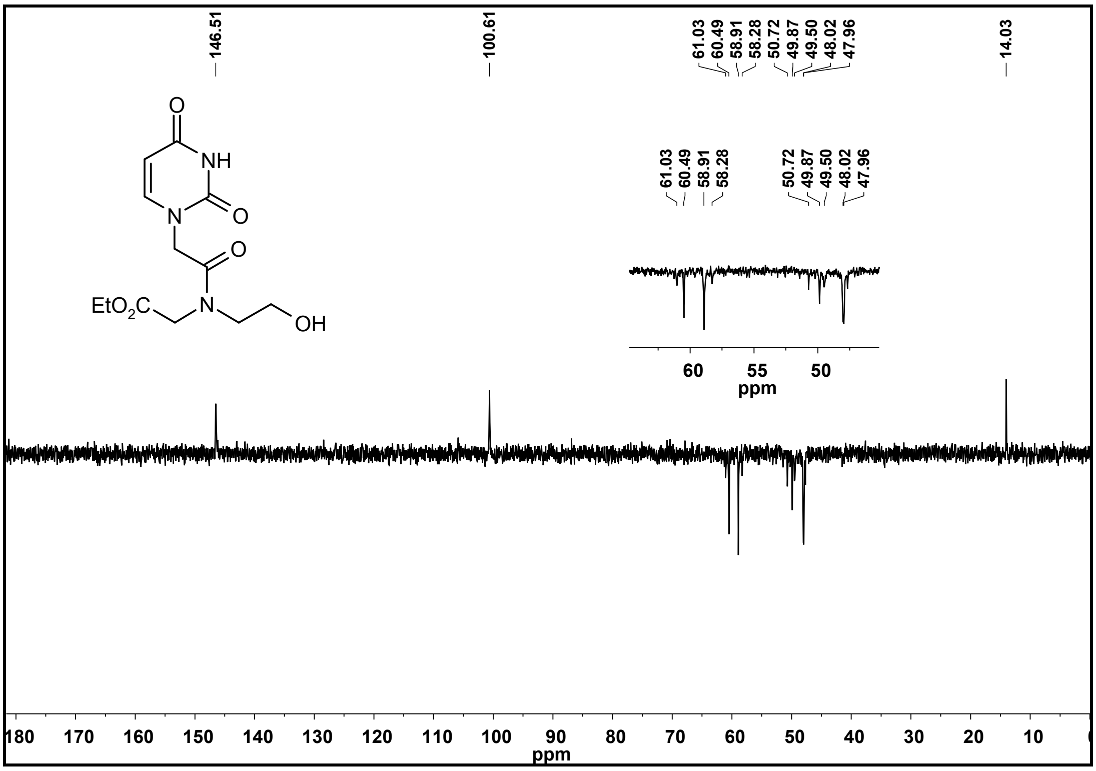

DEPT 135 NMR of compound **7**.

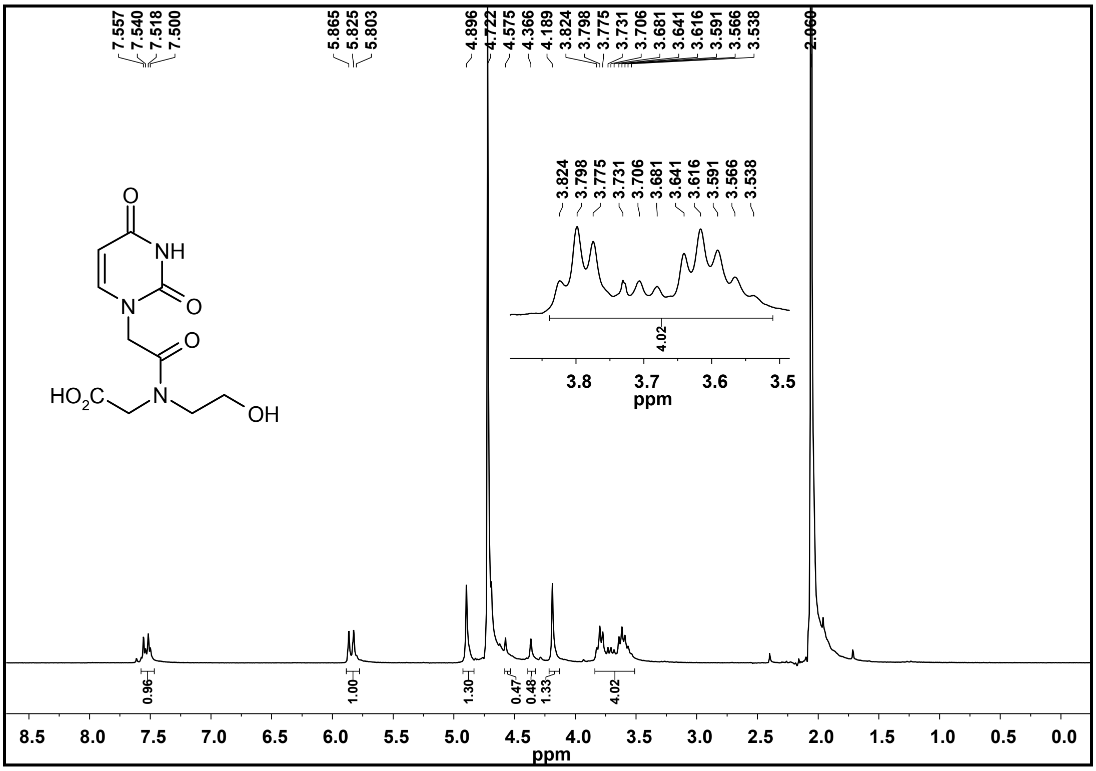

^{1}H-NMR of compound **8**.

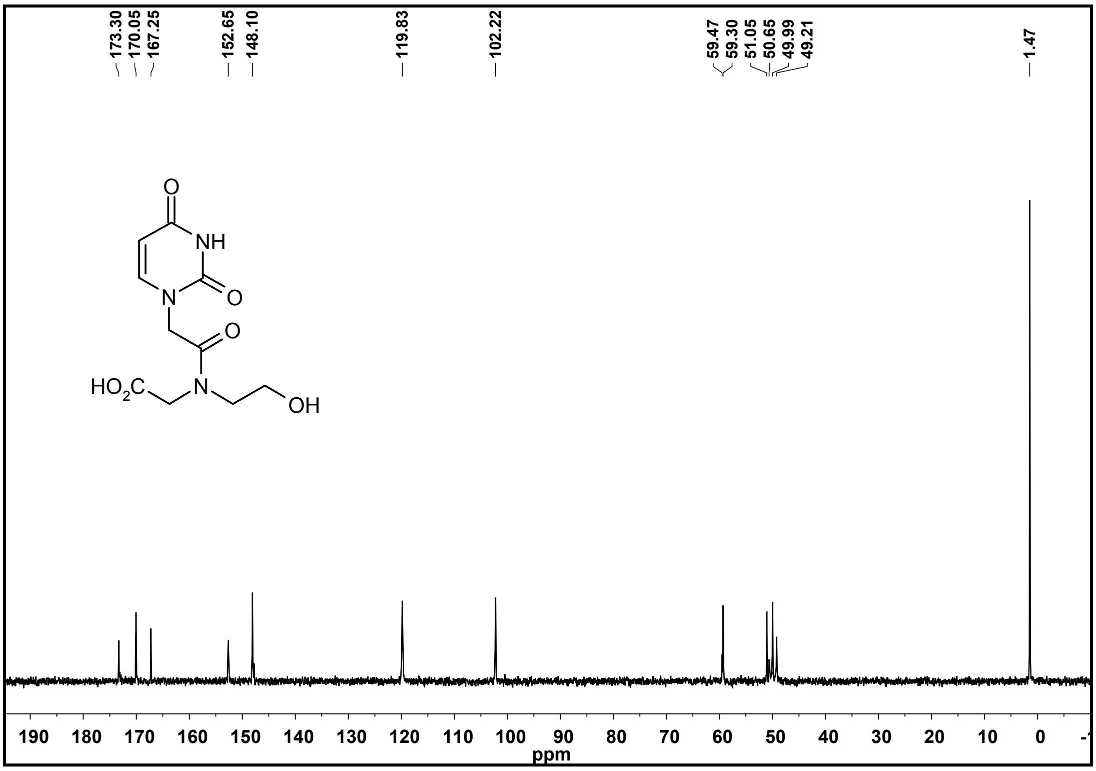

^{13}C-NMR of compound **8**.

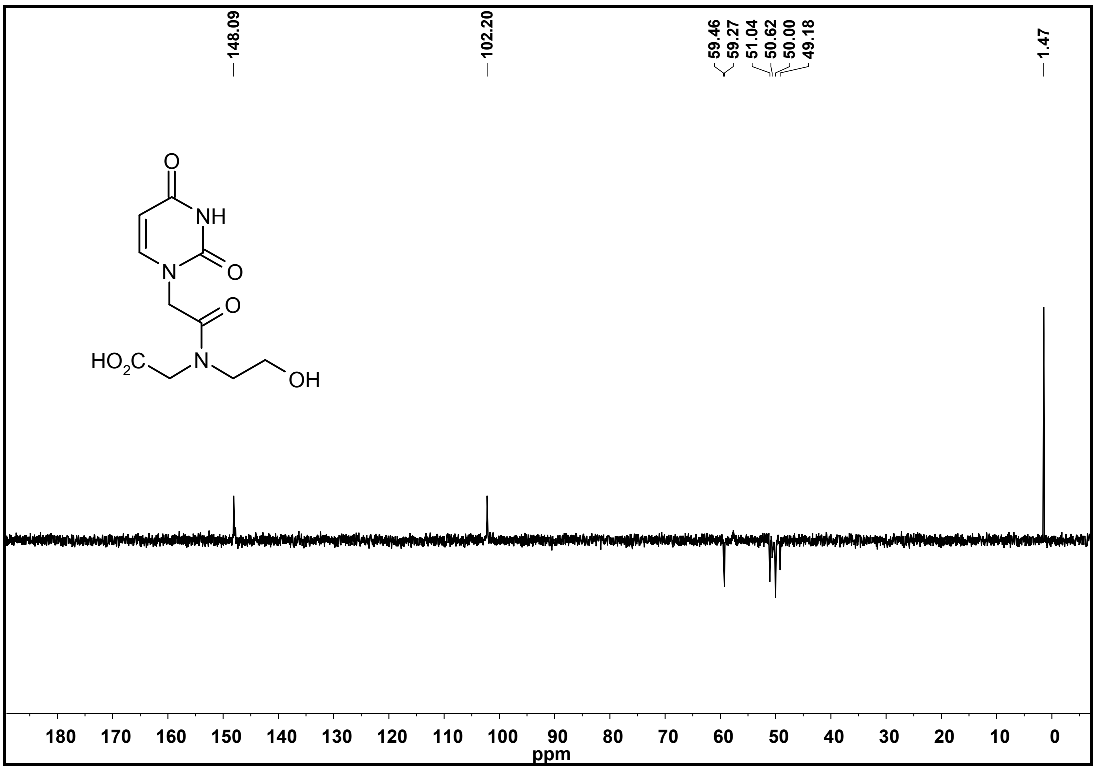

DEPT 135 NMR of compound **8**.

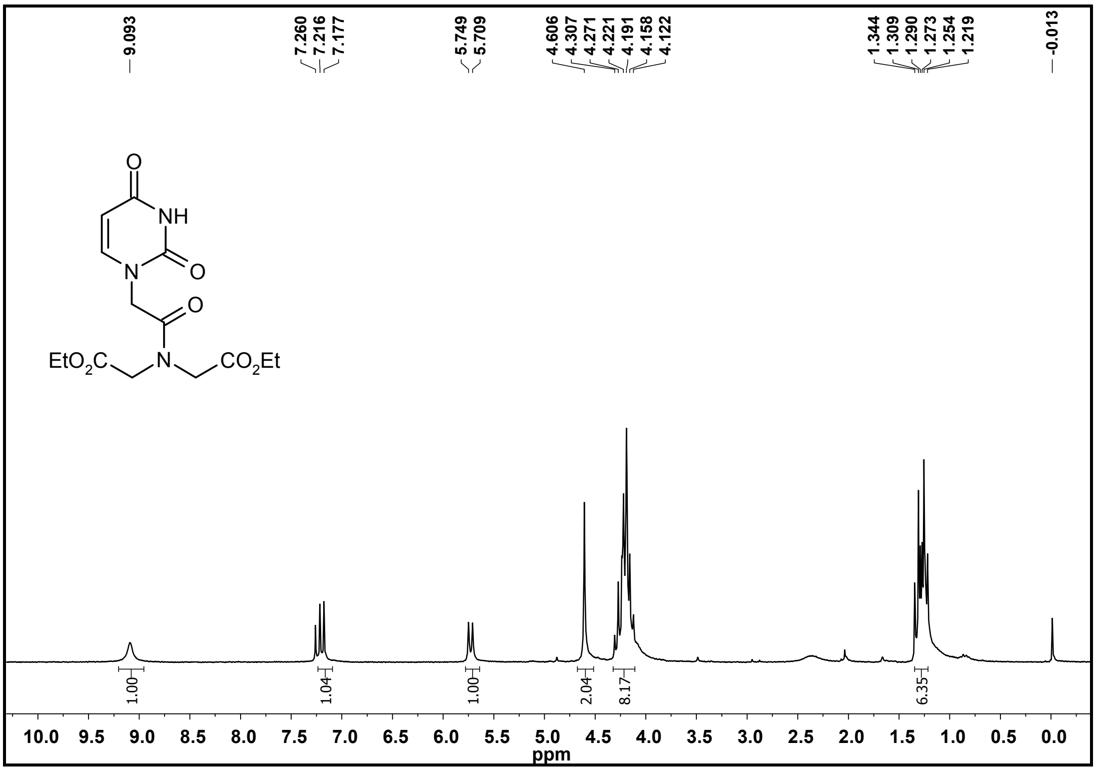

^{1}H-NMR of compound **9**.

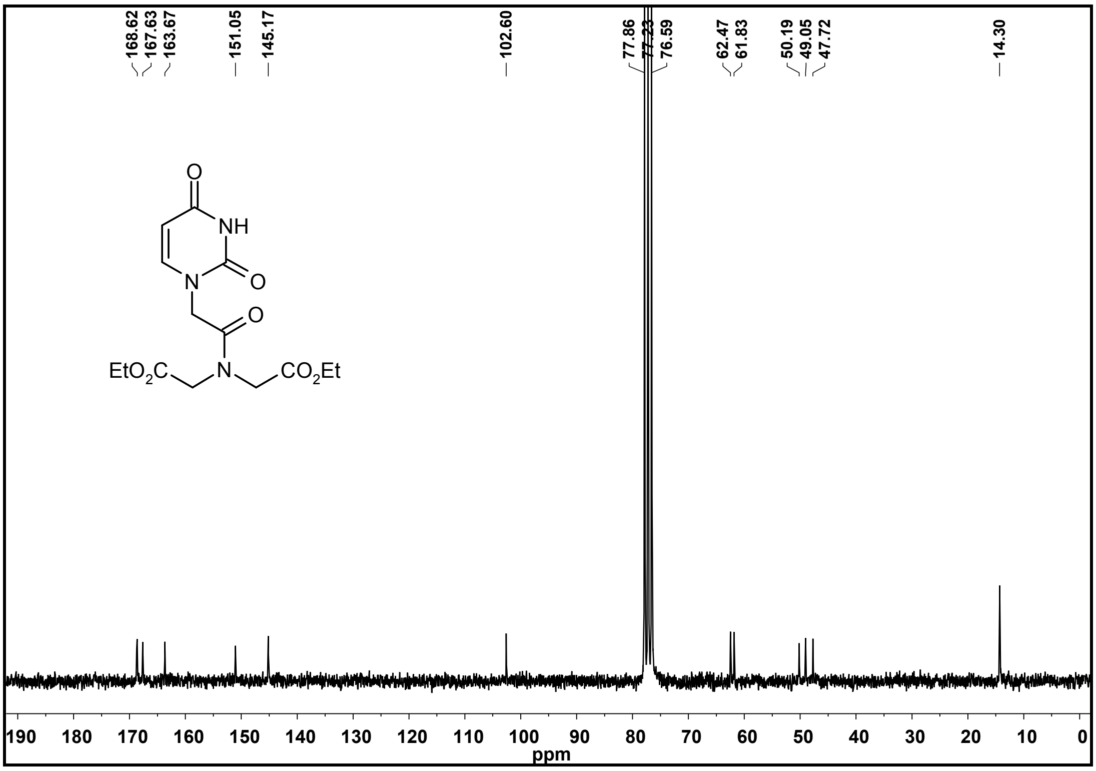

^{13}C-NMR of compound **9**.

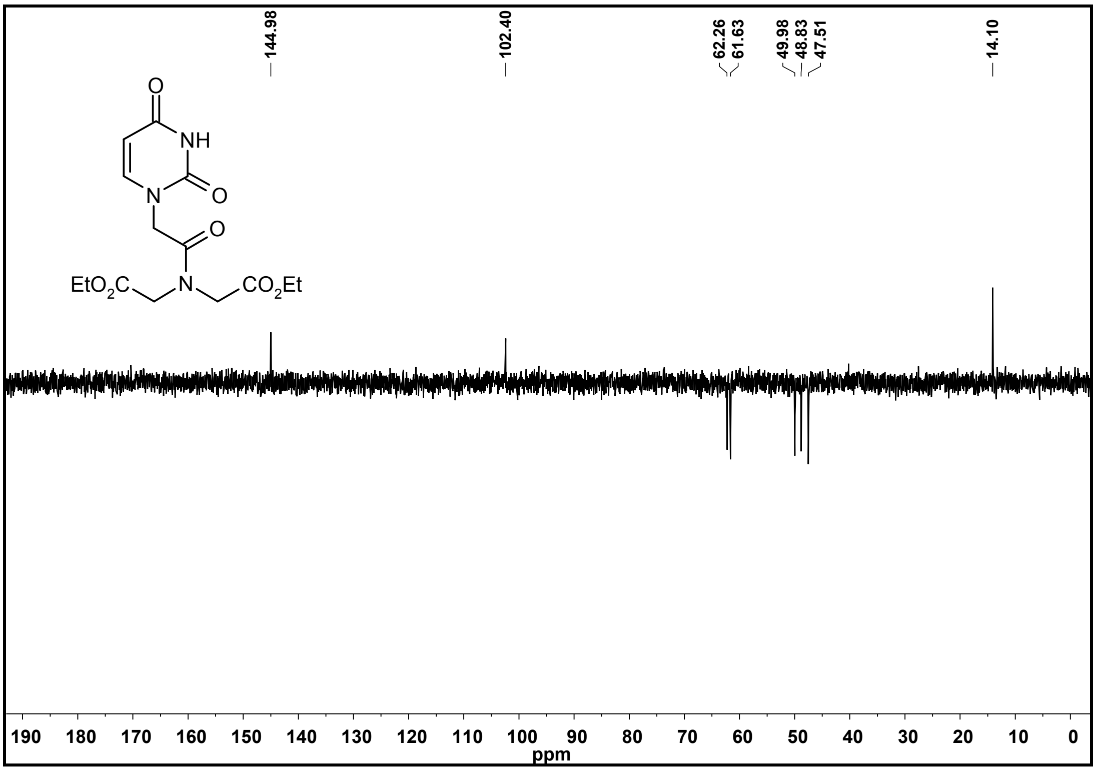

DEPT 135 NMR of compound **9**.

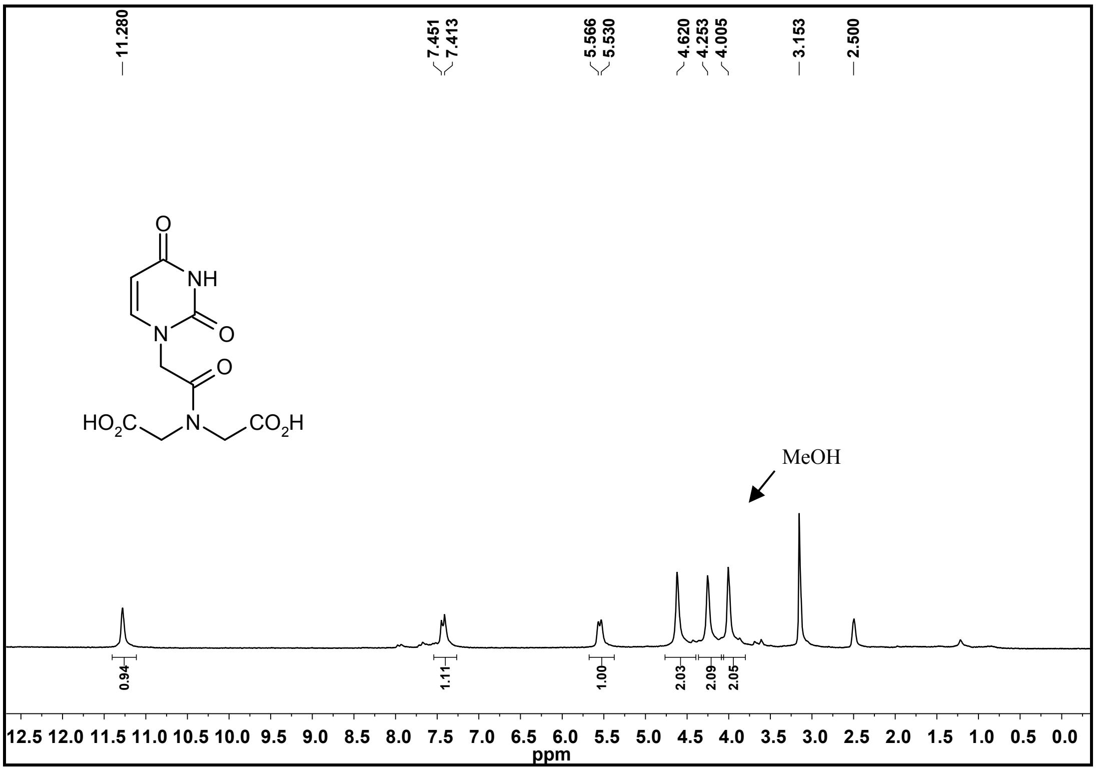

^{1}H-NMR of compound **10**.

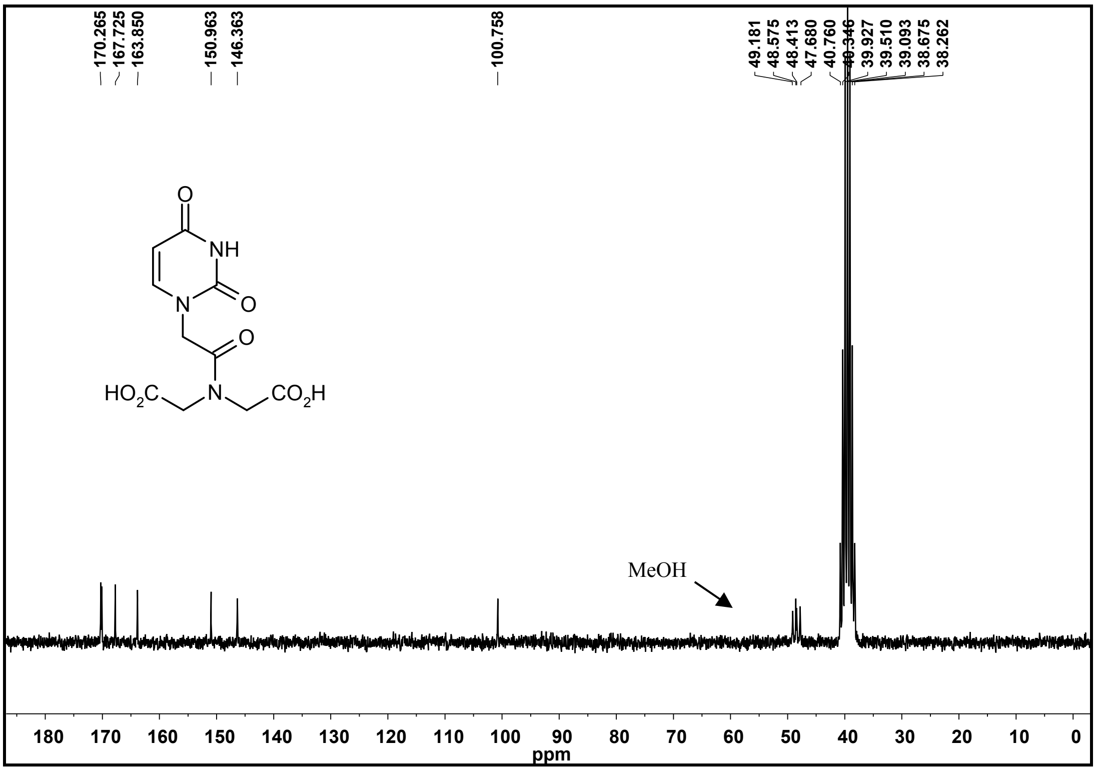

^{13}C-NMR of compound **10**.

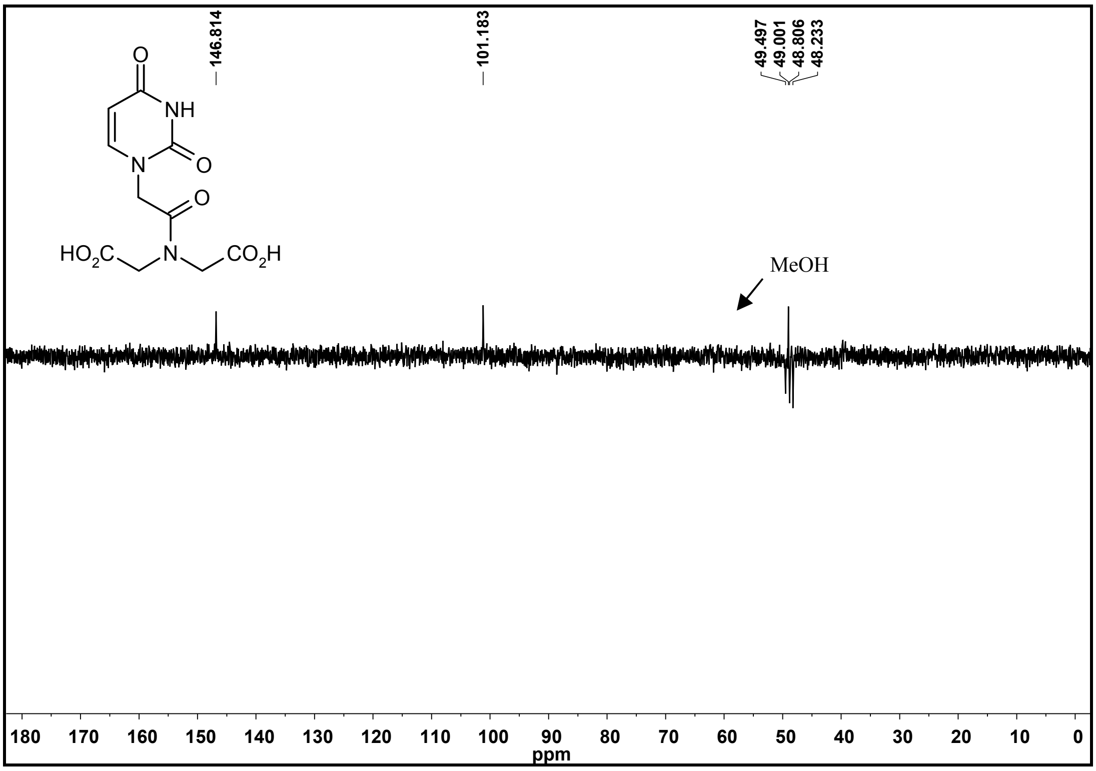

DEPT 135 NMR of compound **10**.

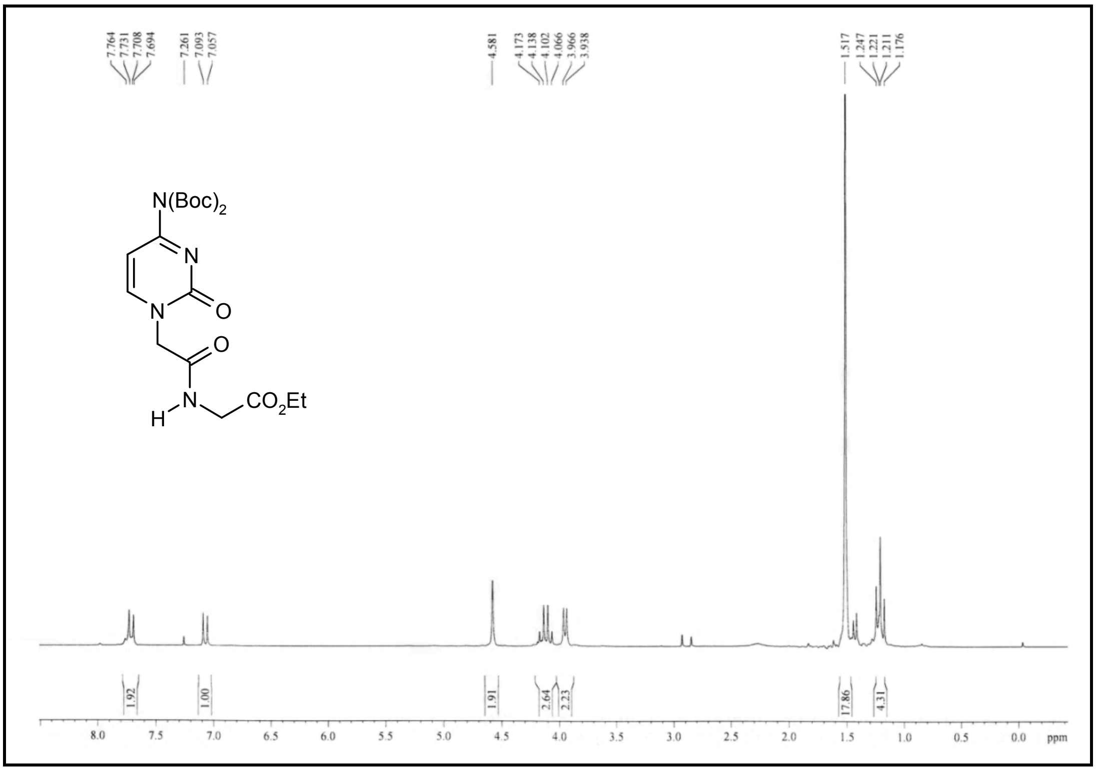

^{1}H-NMR of compound **12**.

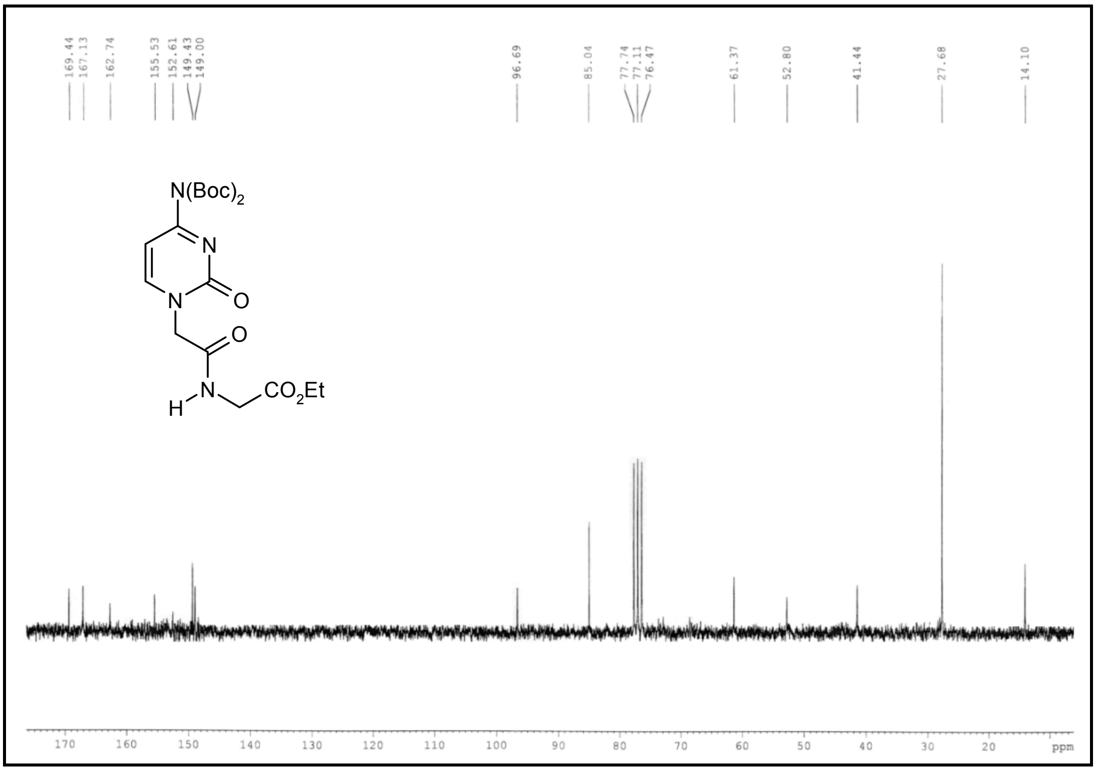

^{13}C-NMR of compound **12**.

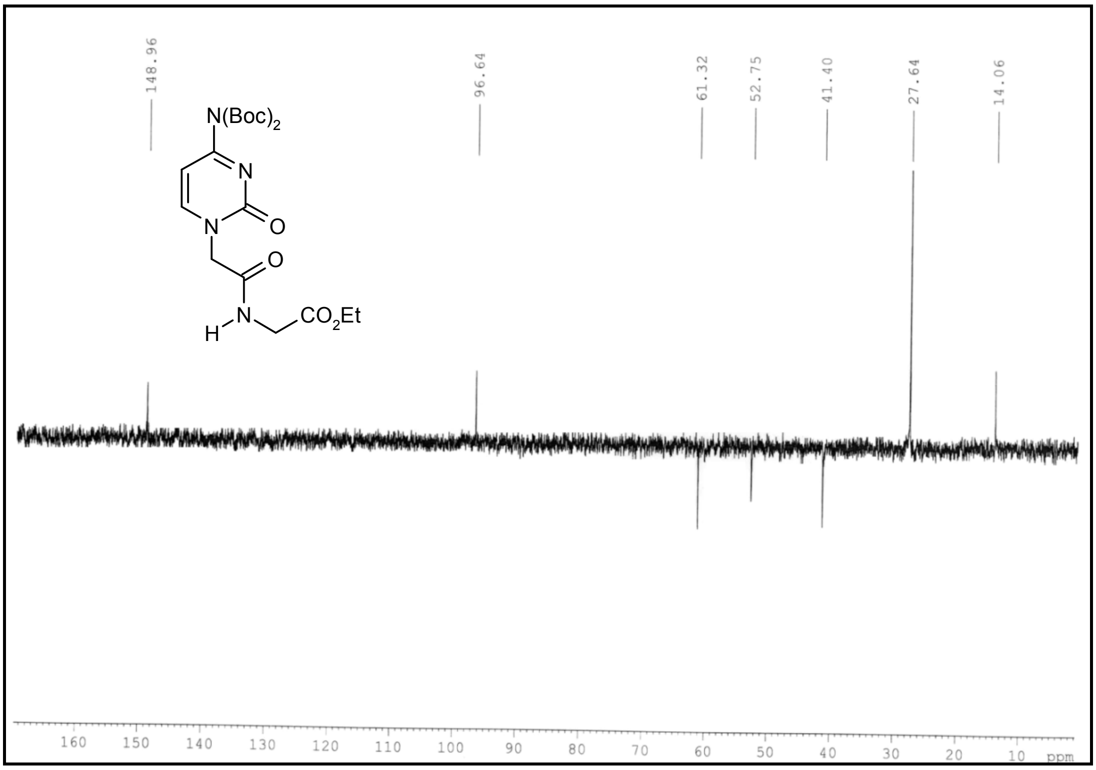

DEPT 135 NMR of compound **12**.

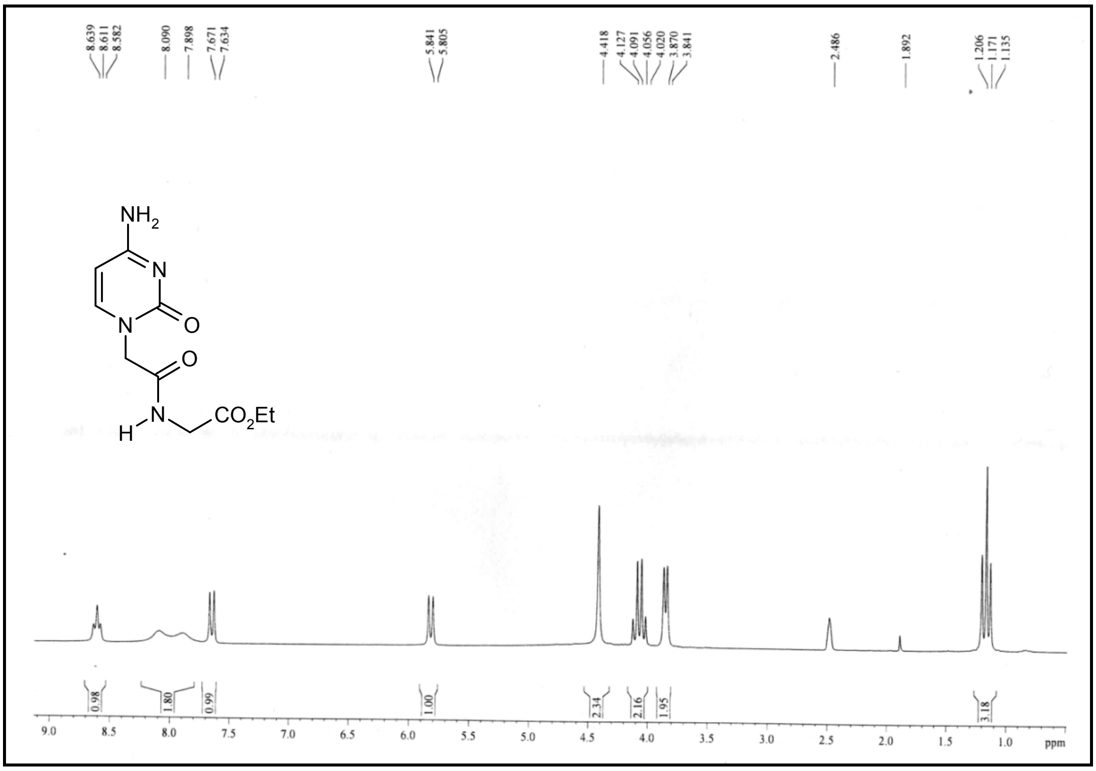

^{1}H-NMR of compound **13**.

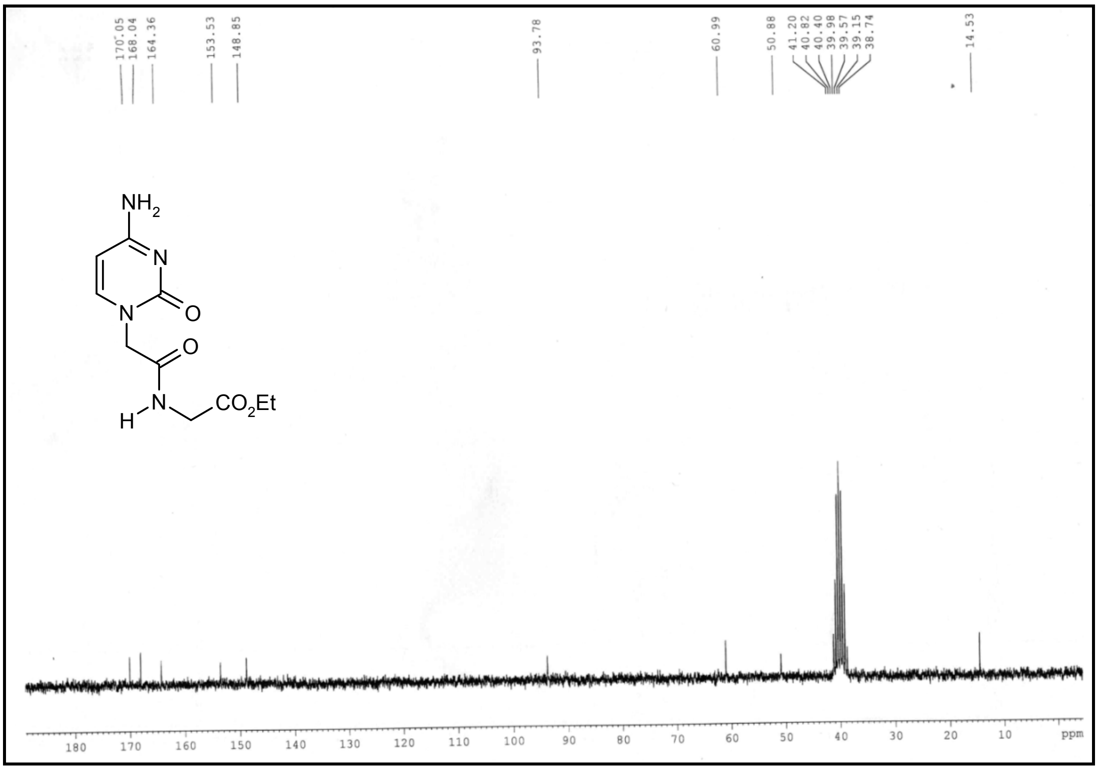

^{13}C-NMR of compound **13**.

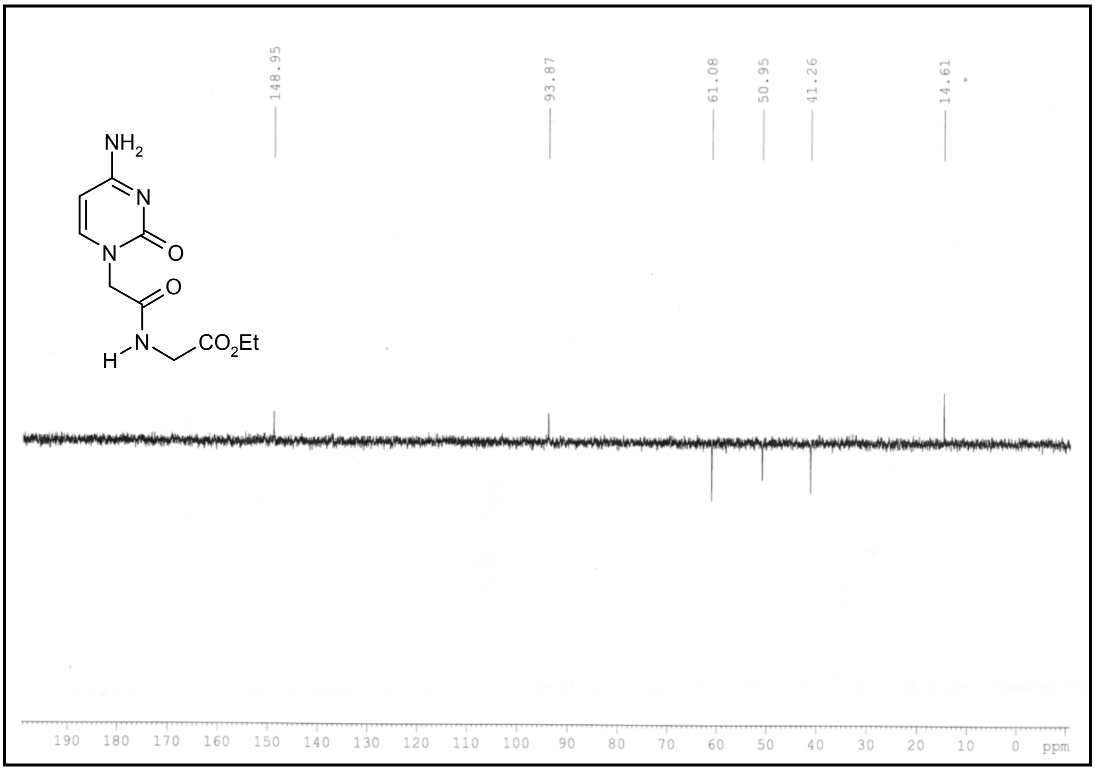

DEPT 135 NMR of compound **13**.

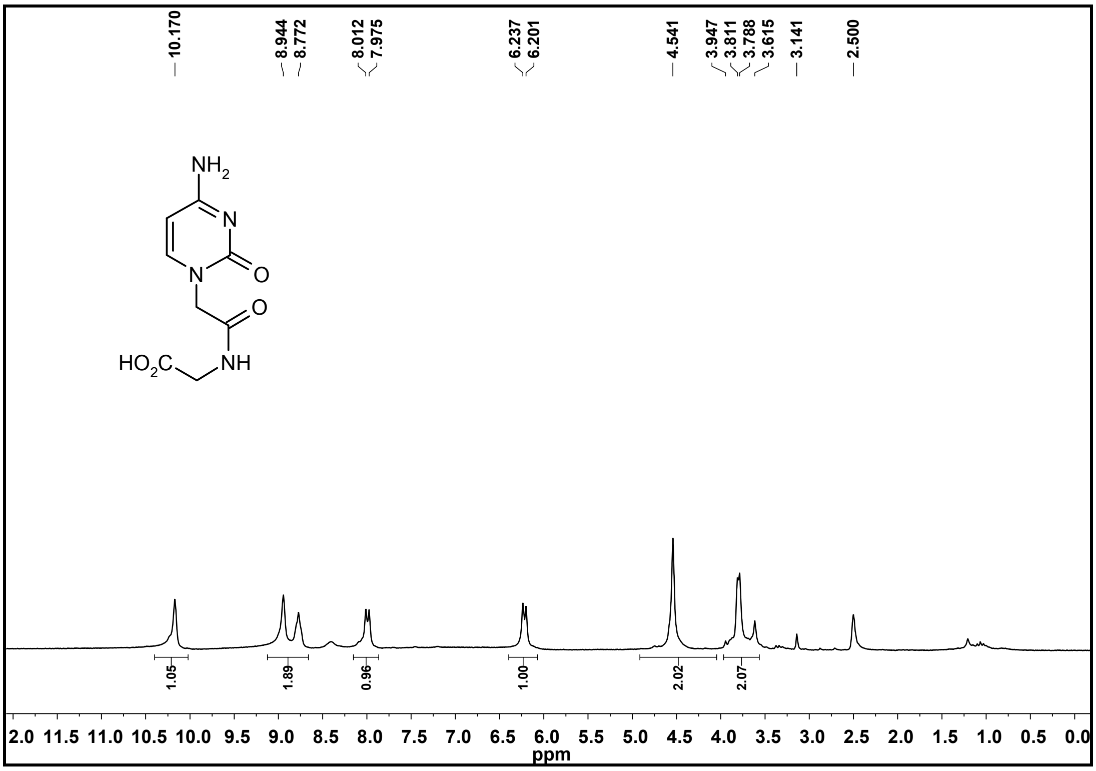

^{1}H-NMR of compound **14**.

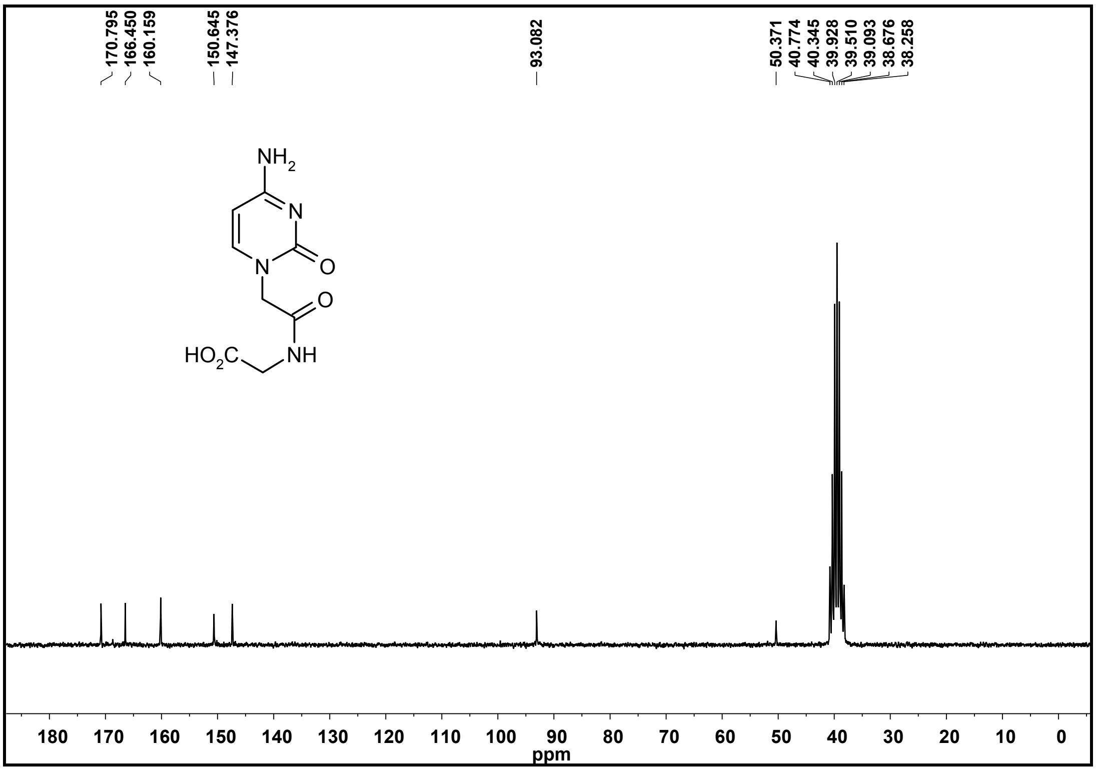

^{13}C-NMR of compound **14**.

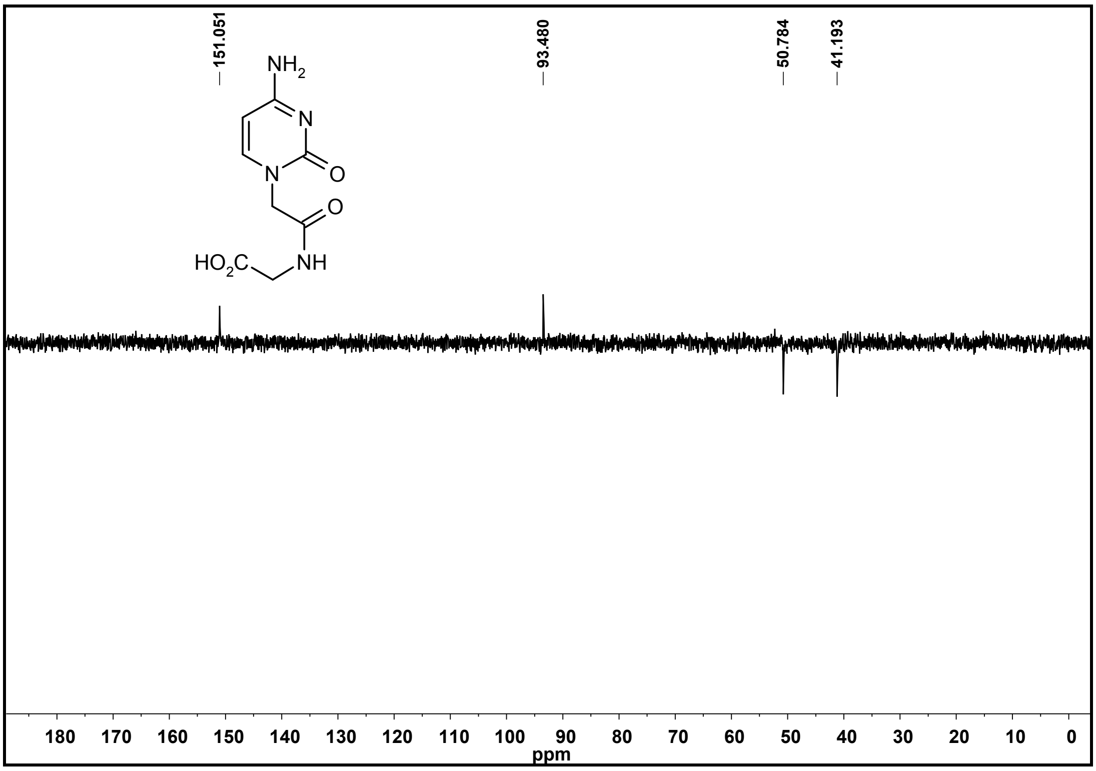

DEPT 135 NMR of compound **14**.

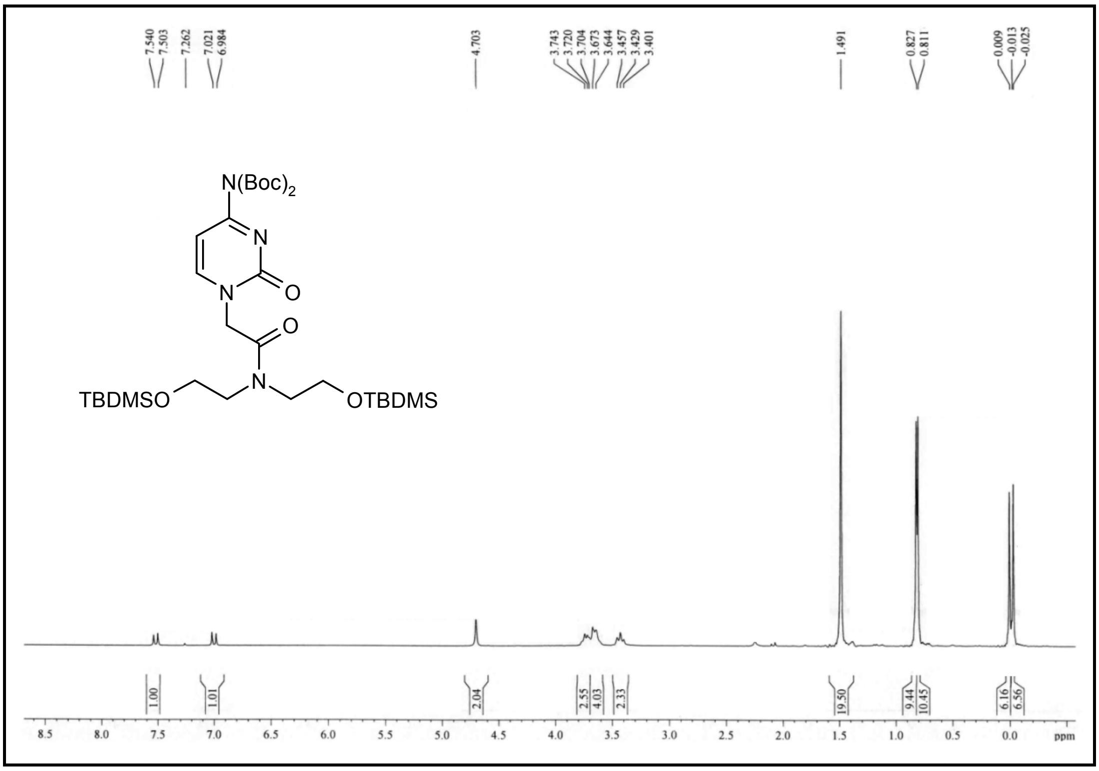

^{1}H-NMR of compound **15**.

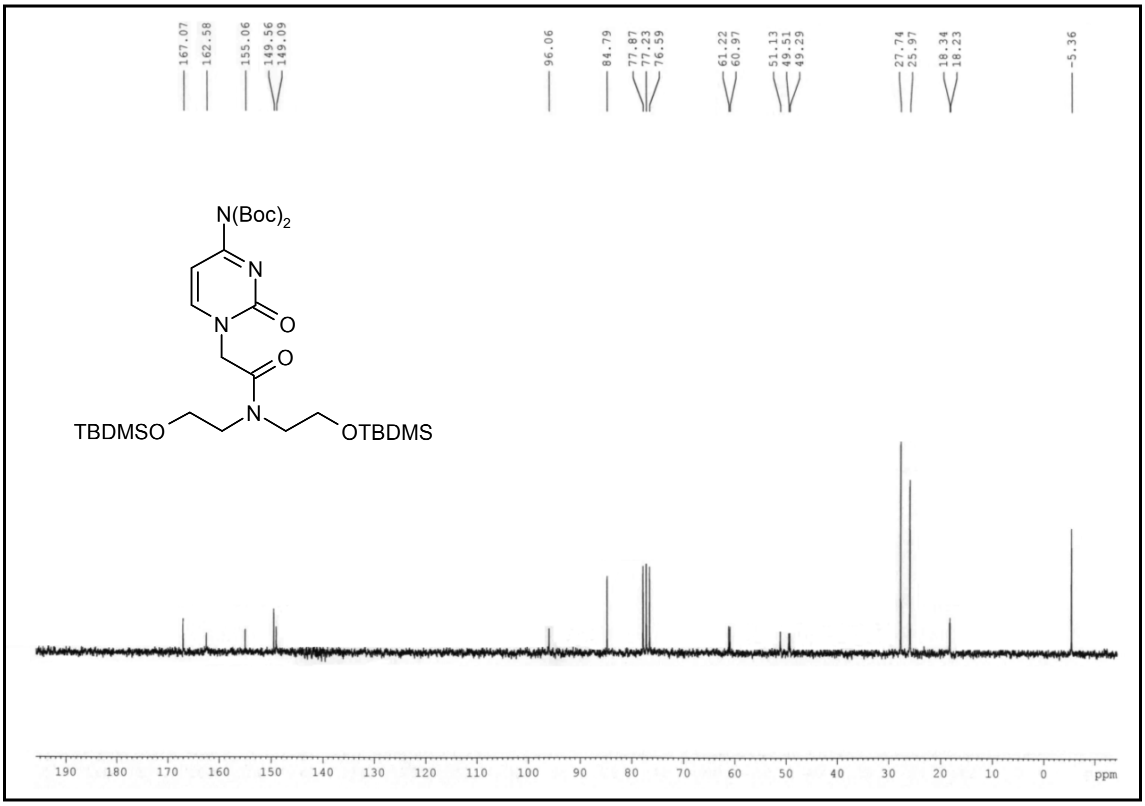

^{13}C-NMR of compound **15**.

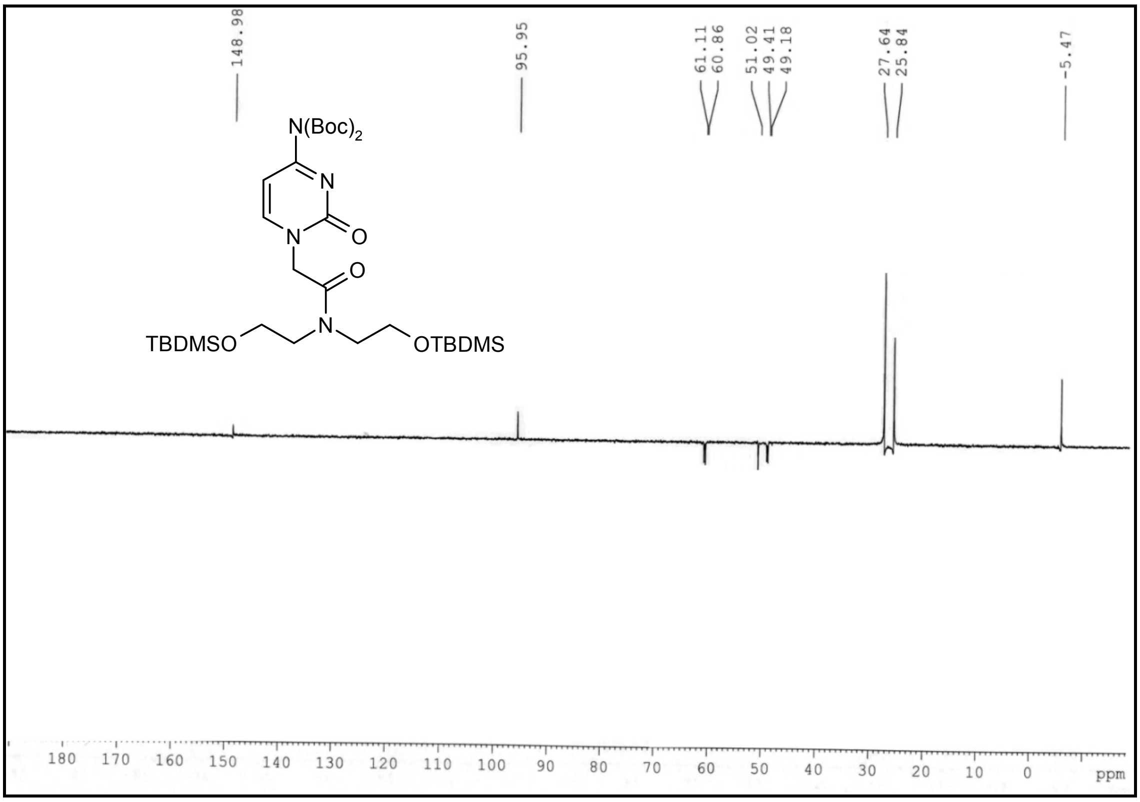

DEPT 135 NMR of compound **15**.

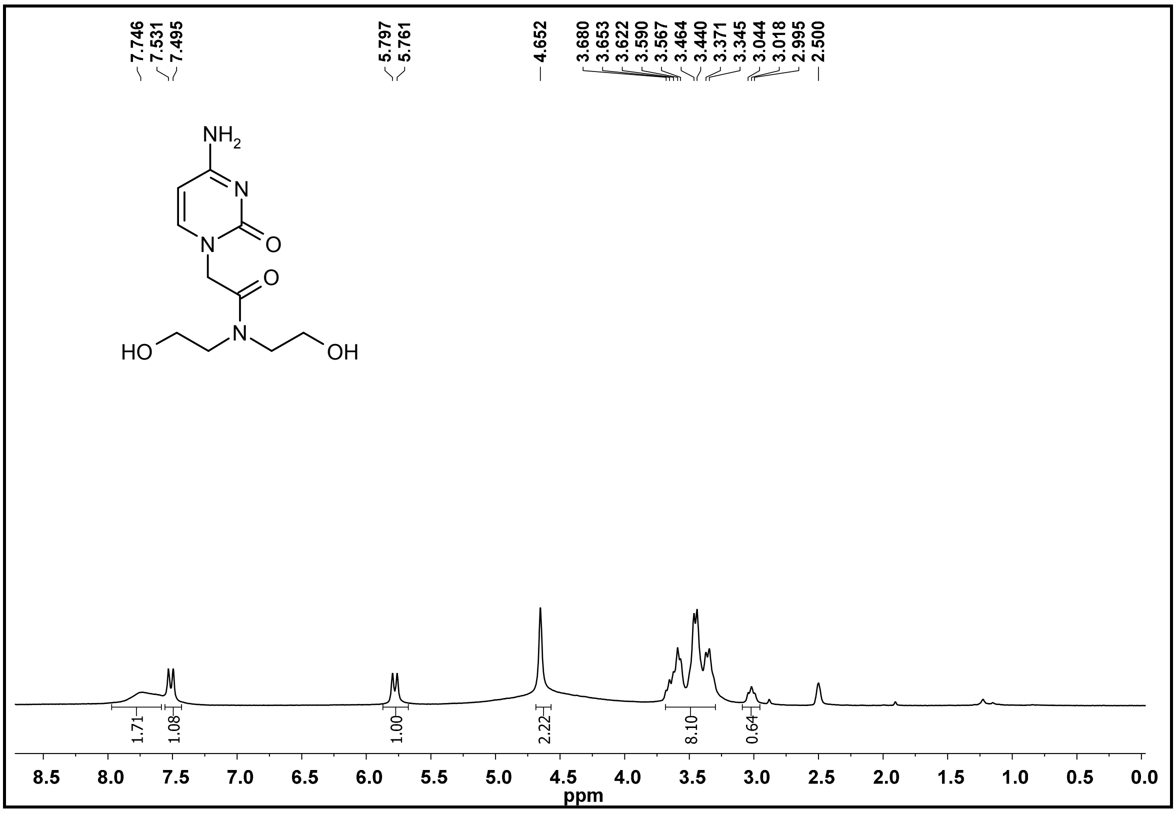

^{1}H-NMR of compound **16**.

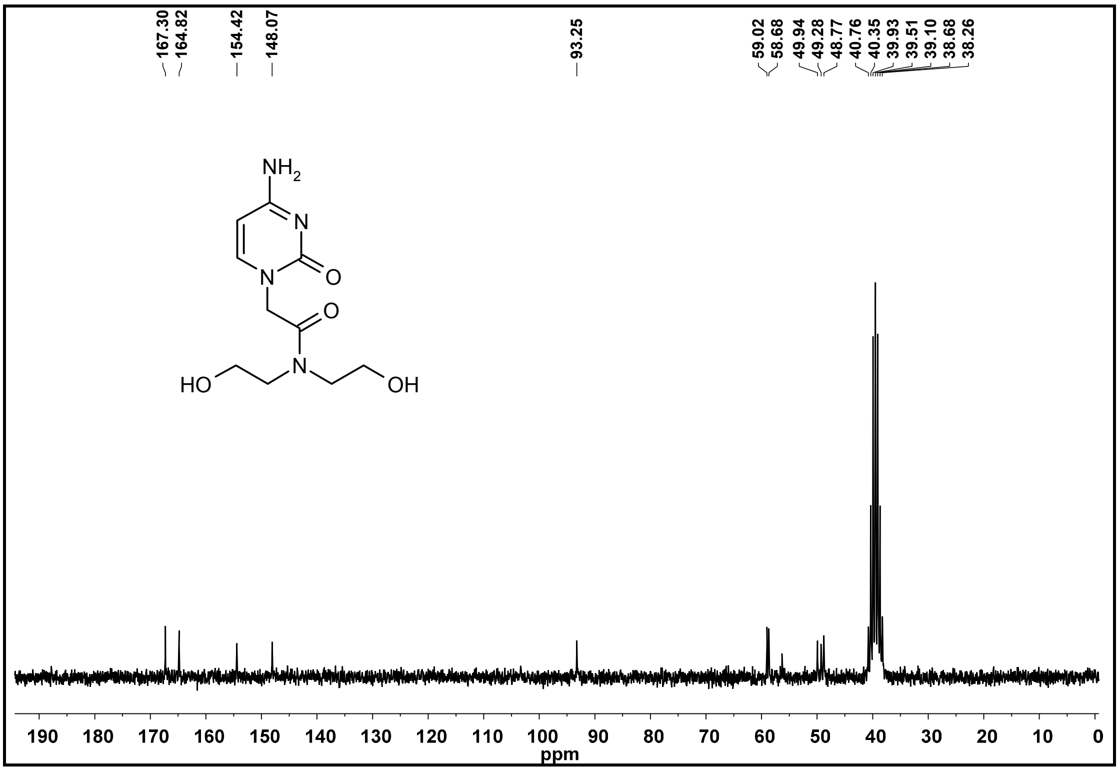

^{13}C-NMR of compound **16**.

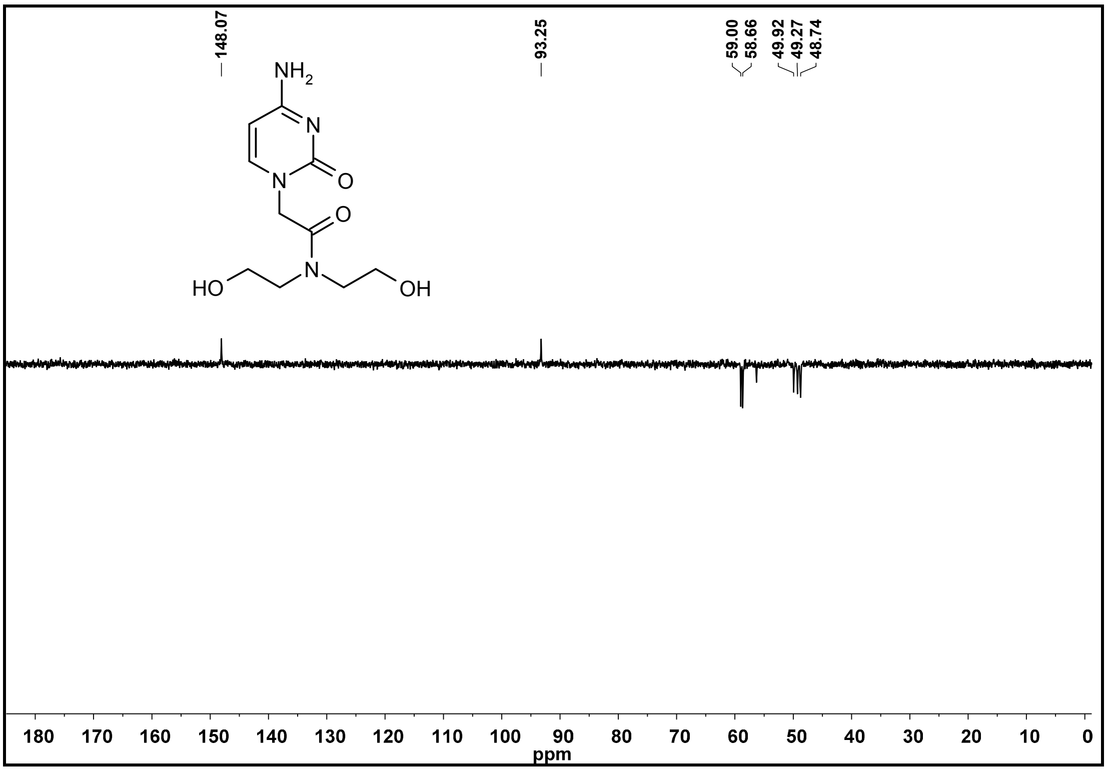

DEPT 135 NMR of compound **16**.

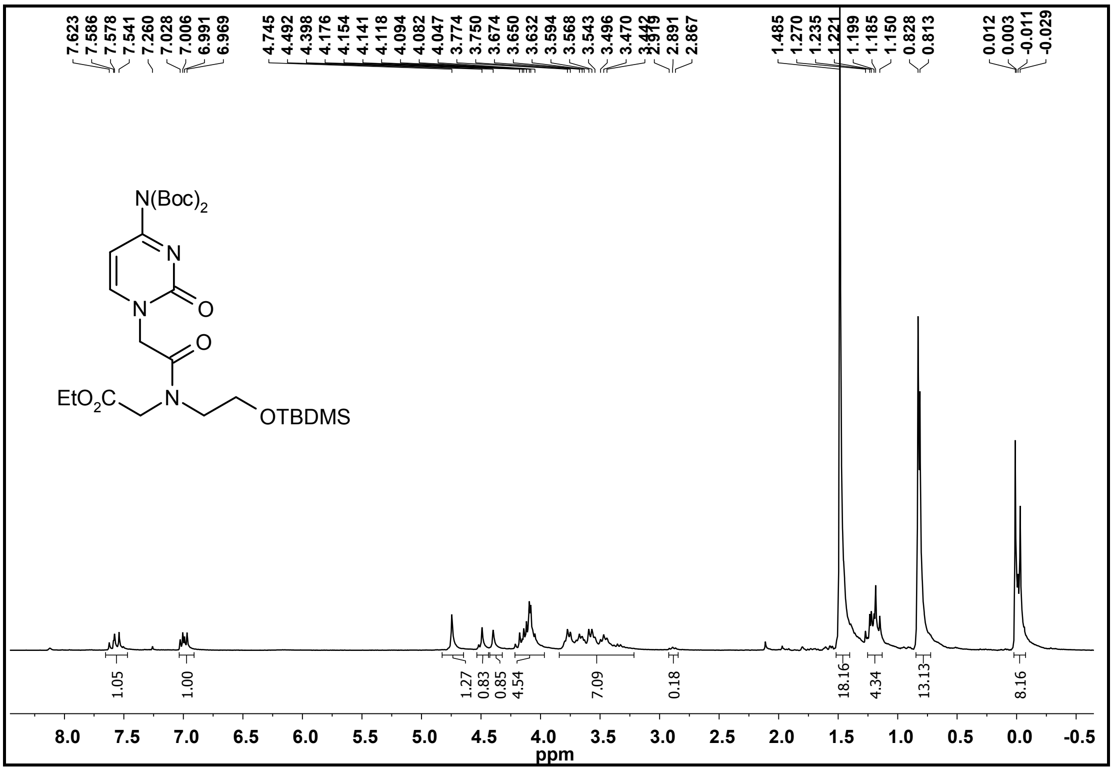

^{1}H-NMR of compound **17**.

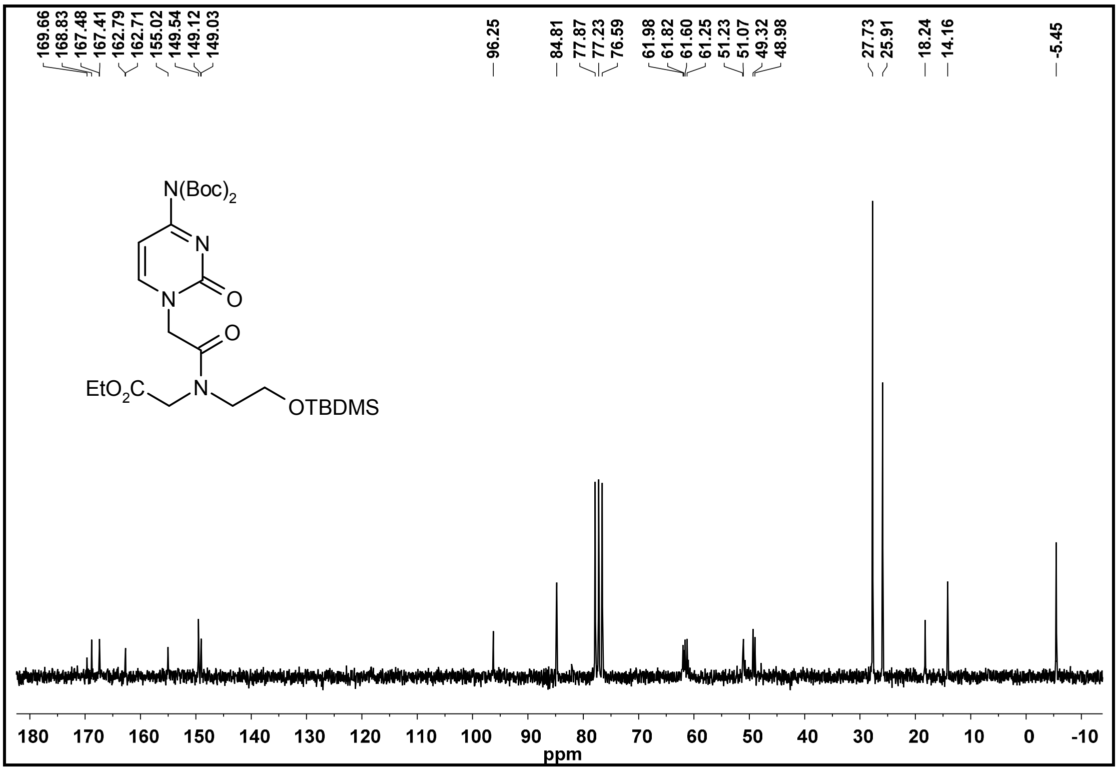

^{13}C-NMR of compound **17**.

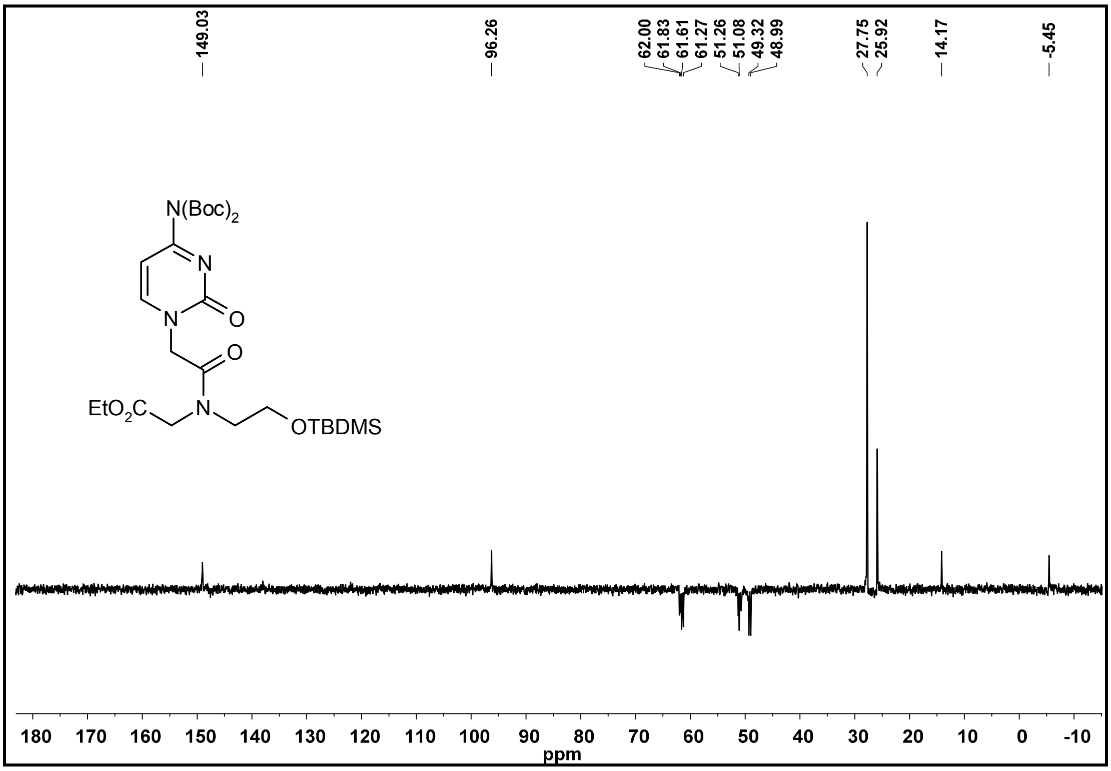

DEPT 135 NMR of compound **17**.

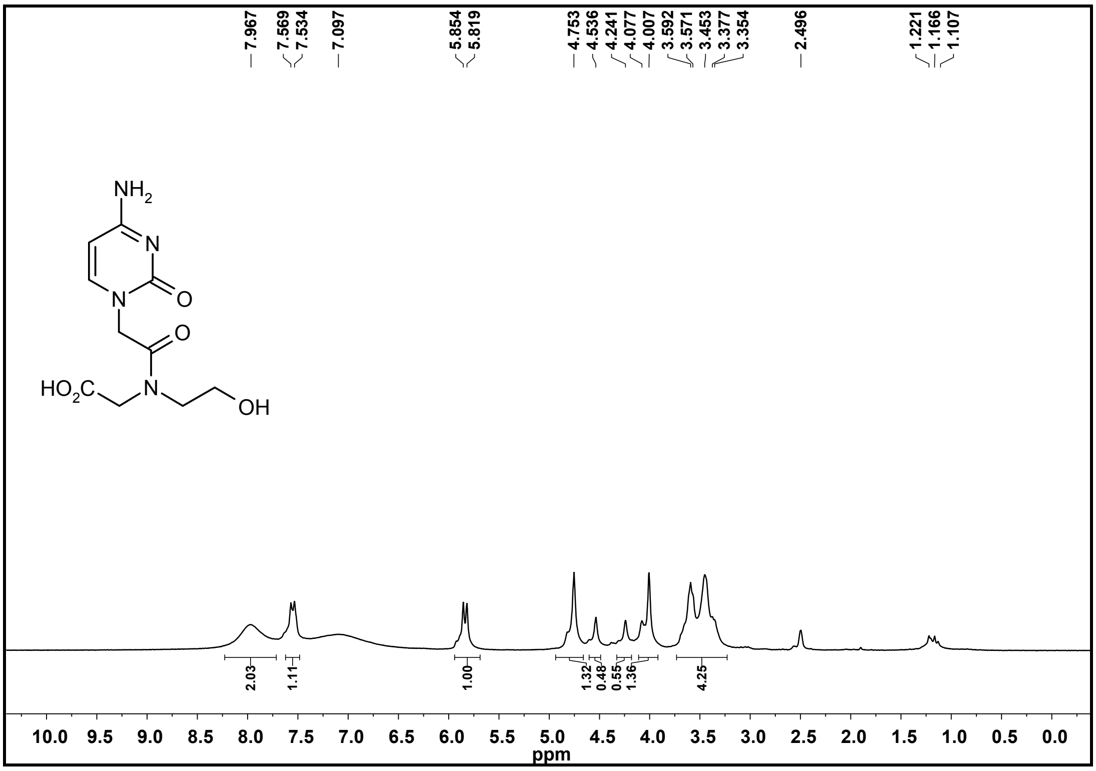

^{1}H-NMR of compound **18**.

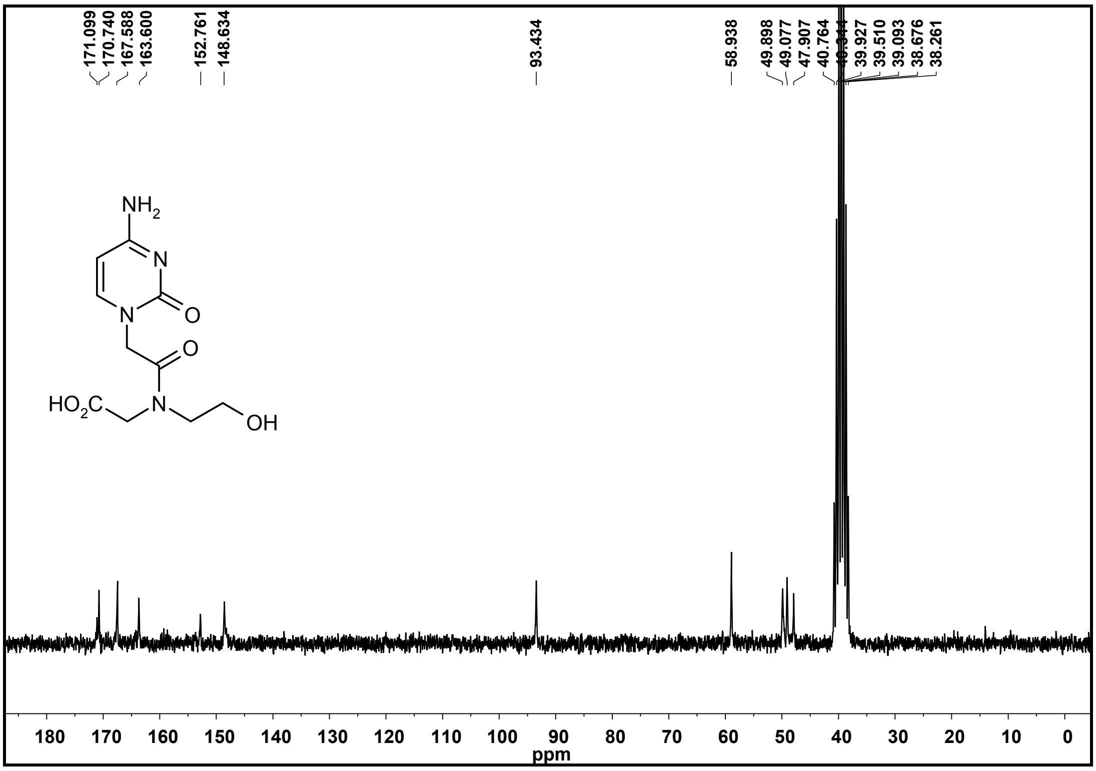

^{13}C-NMR of compound **18**.

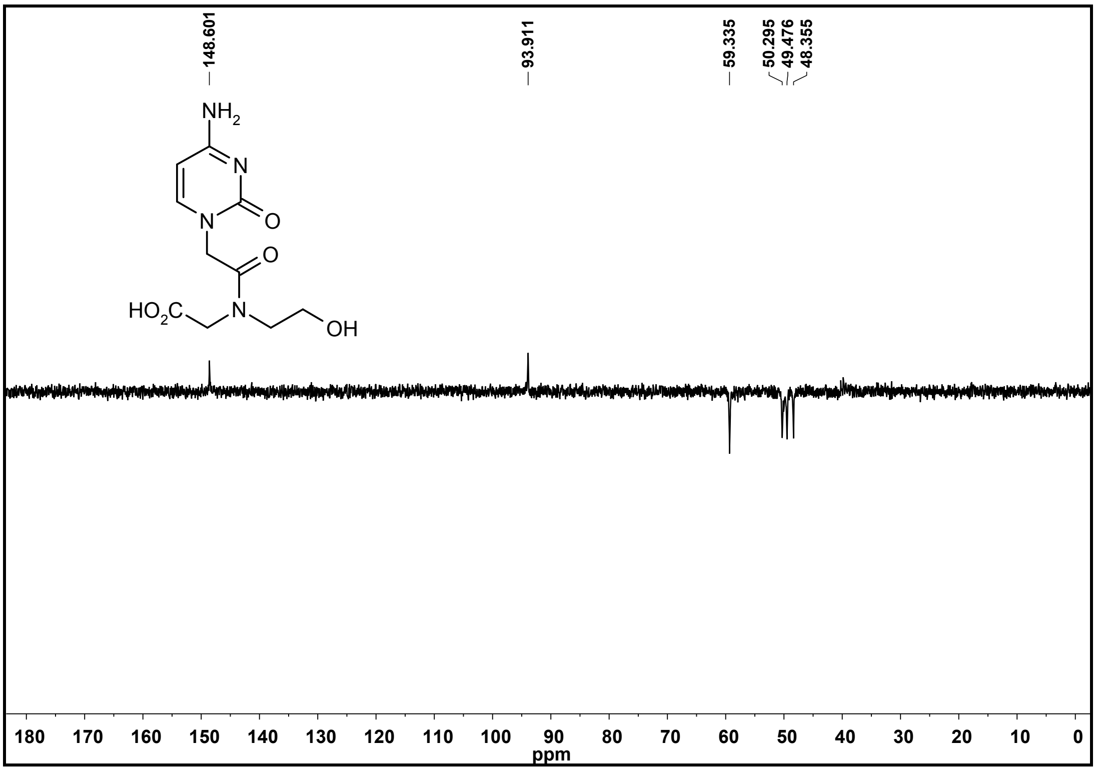

DEPT 135 NMR of compound **18**.

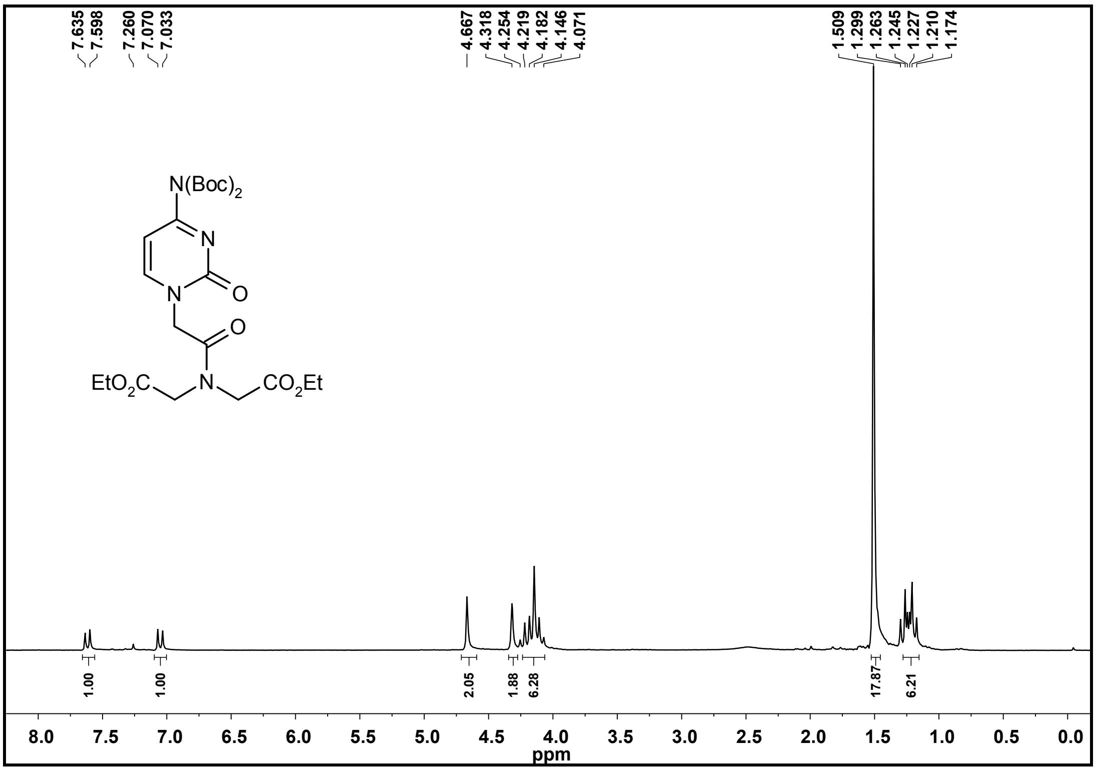

^{1}H-NMR of compound **19**.

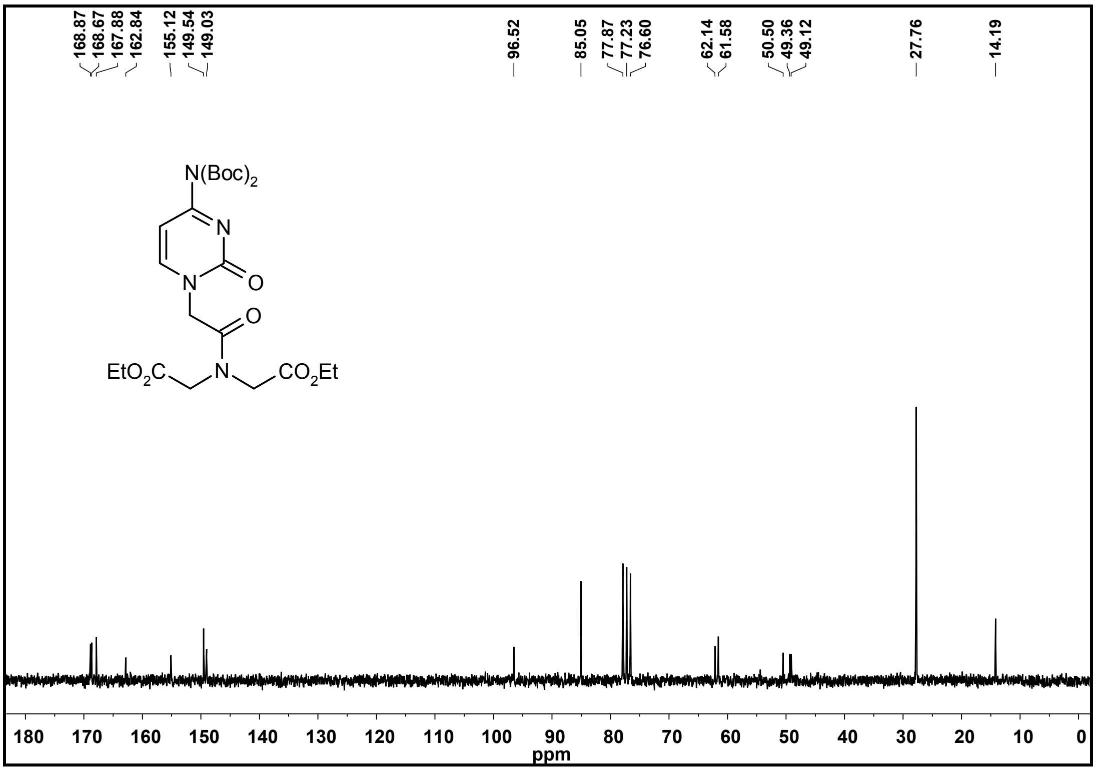

^{13}C-NMR of compound **19**.

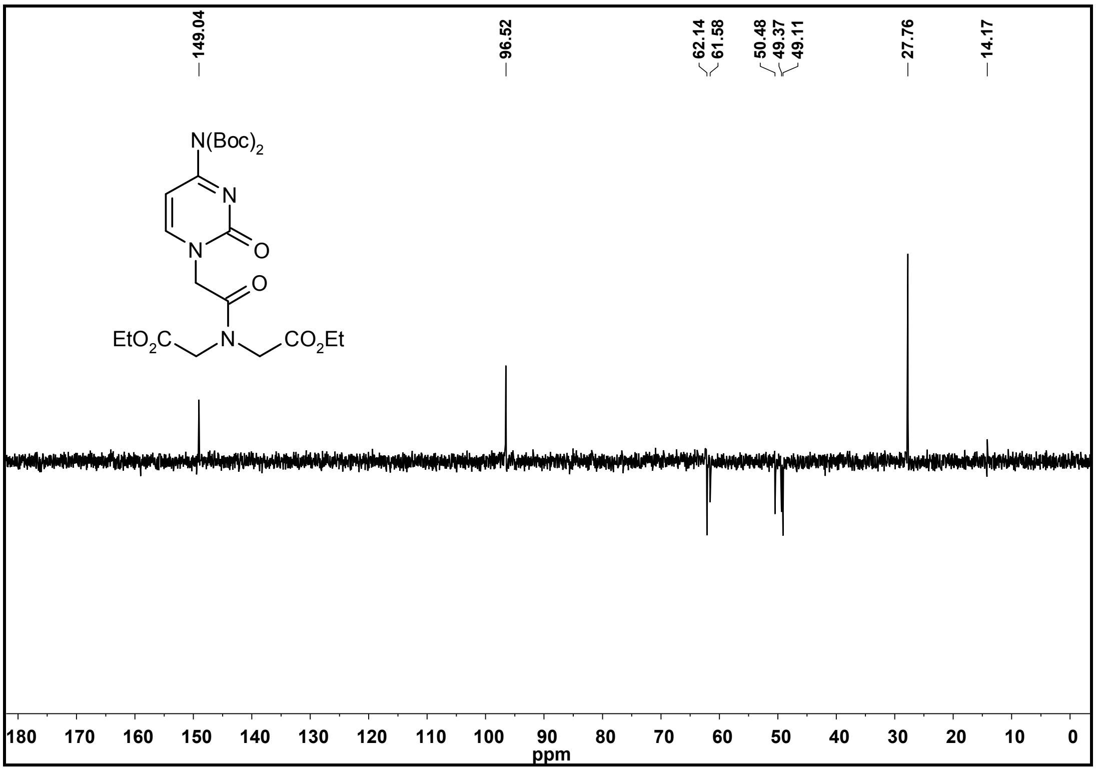

DEPT 135 NMR of compound **19**.

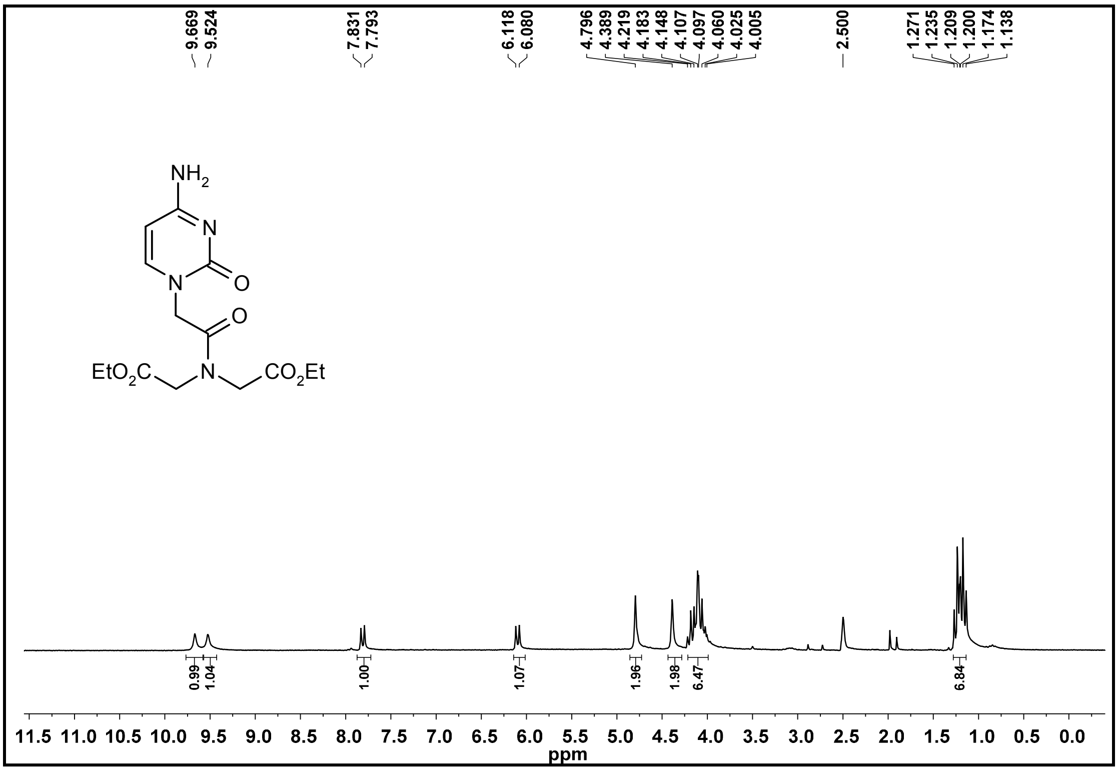

^{1}H-NMR of compound **20**.

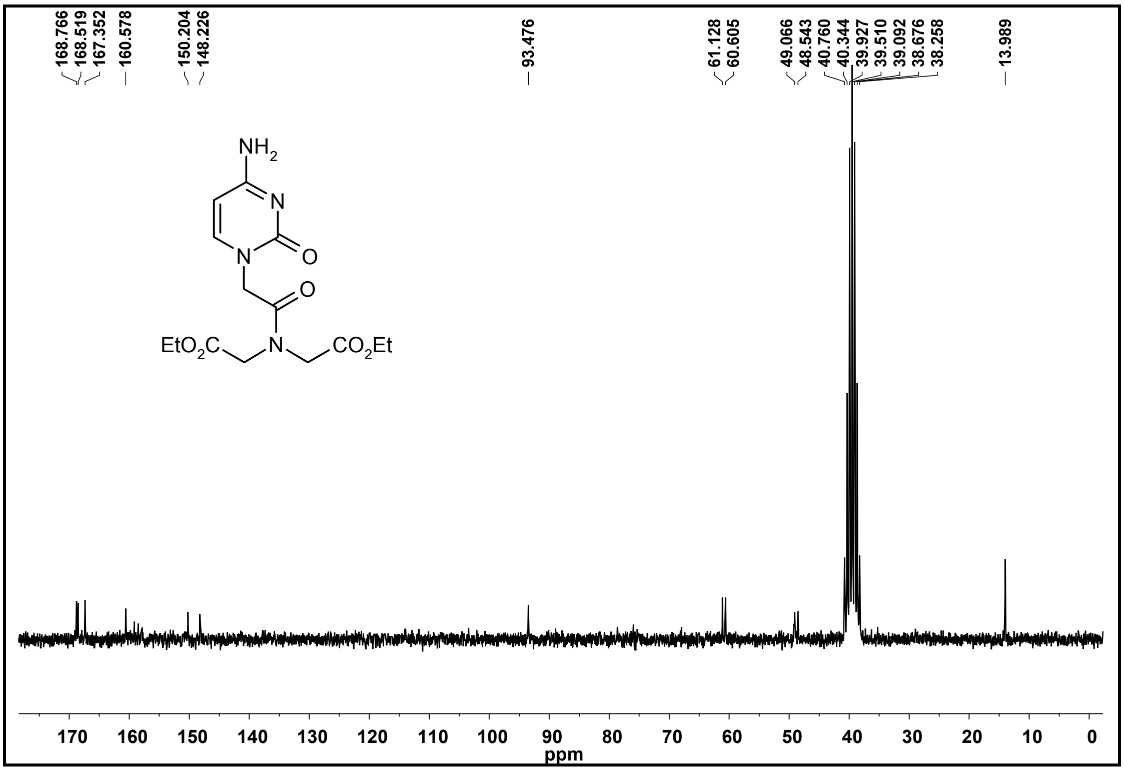

^{13}C-NMR of compound **20**.

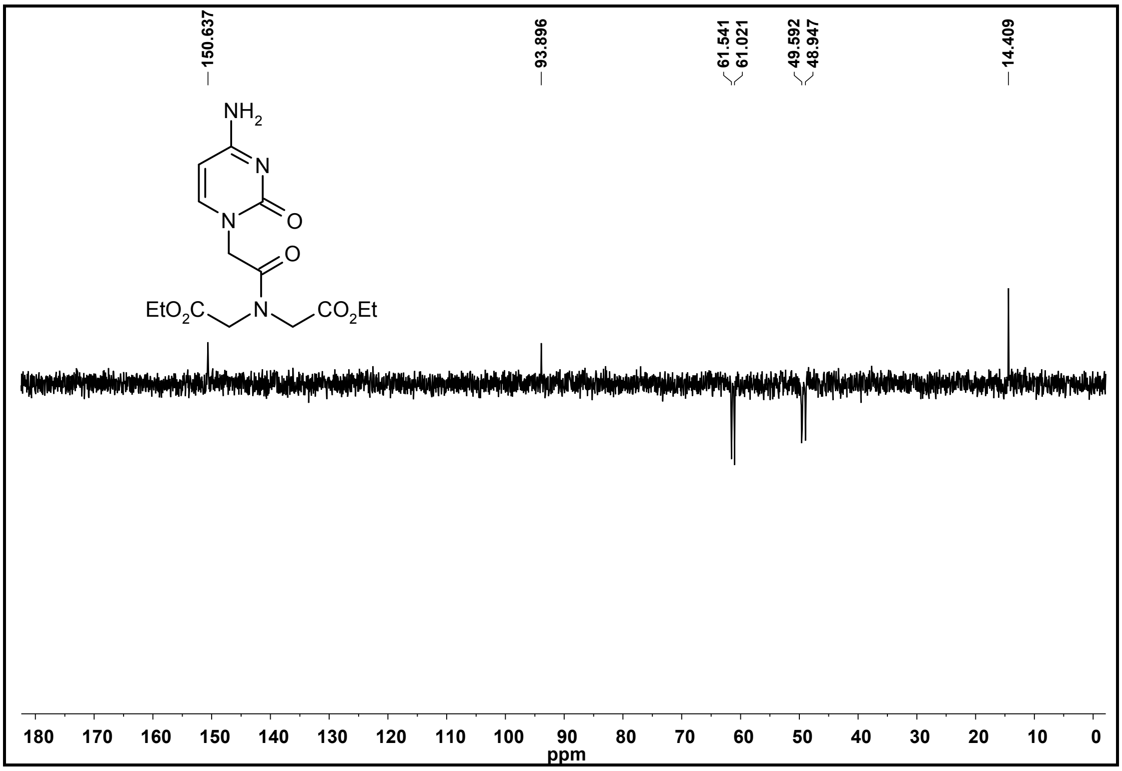

DEPT 135 NMR of compound **20**.

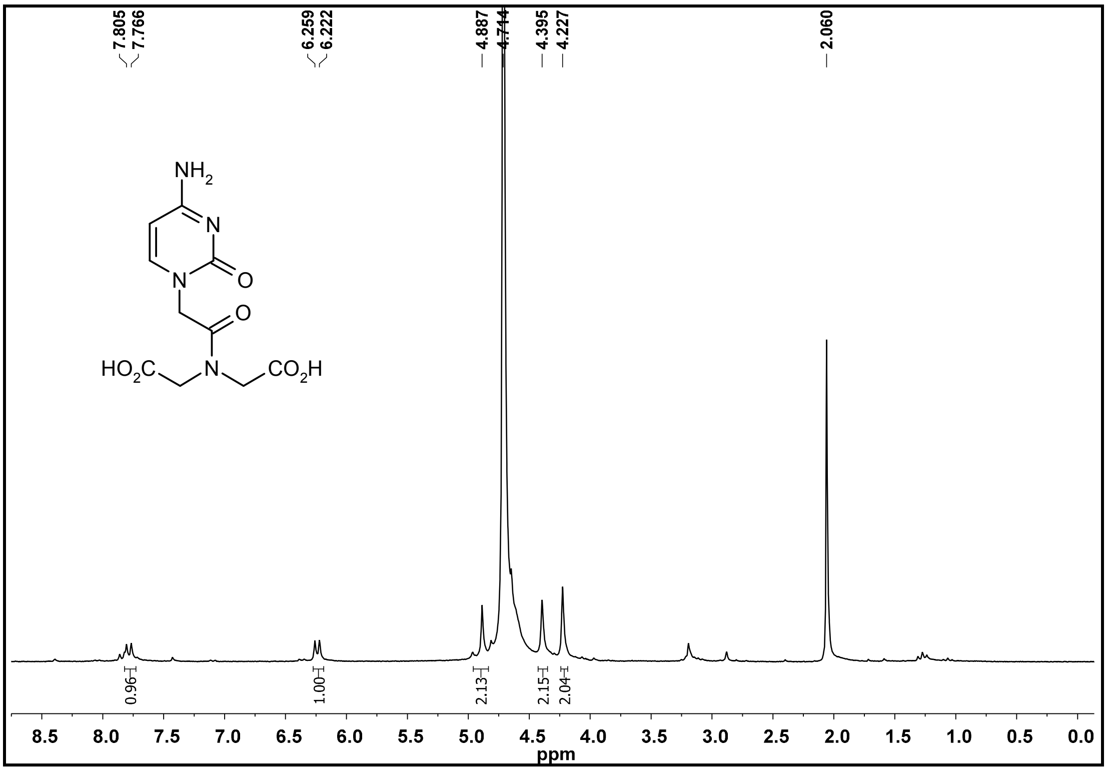

^{1}H-NMR of compound **21**.

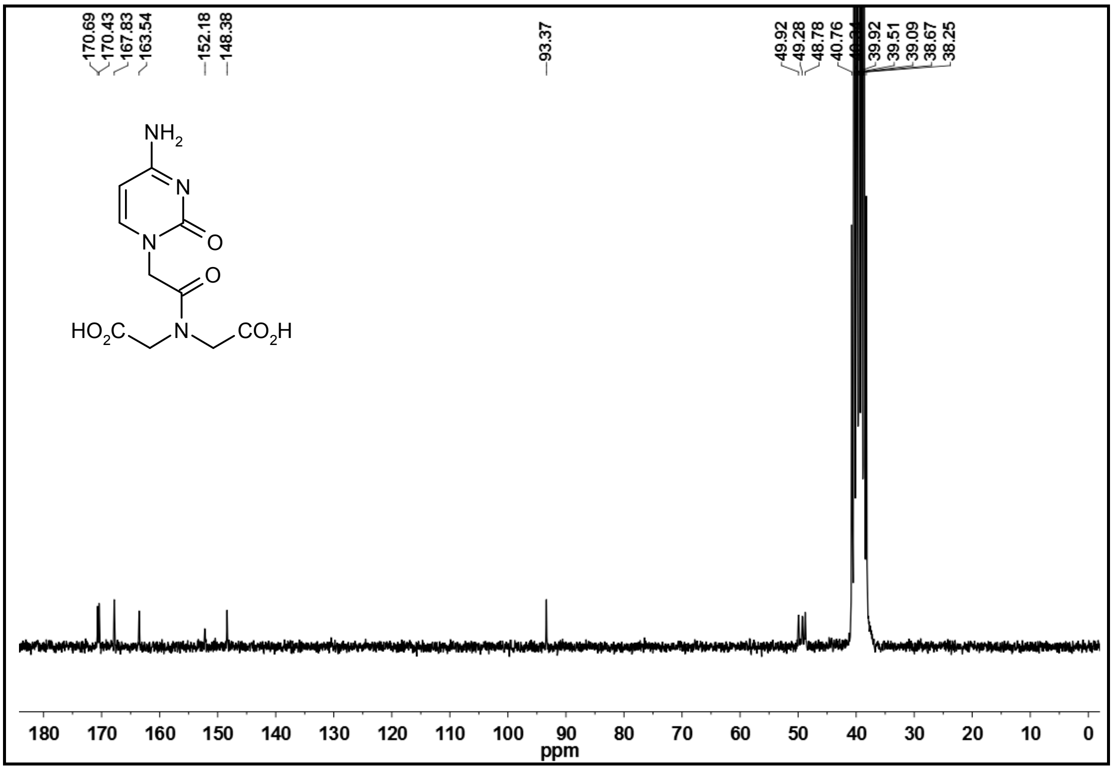

^{13}C-NMR of compound **21**.

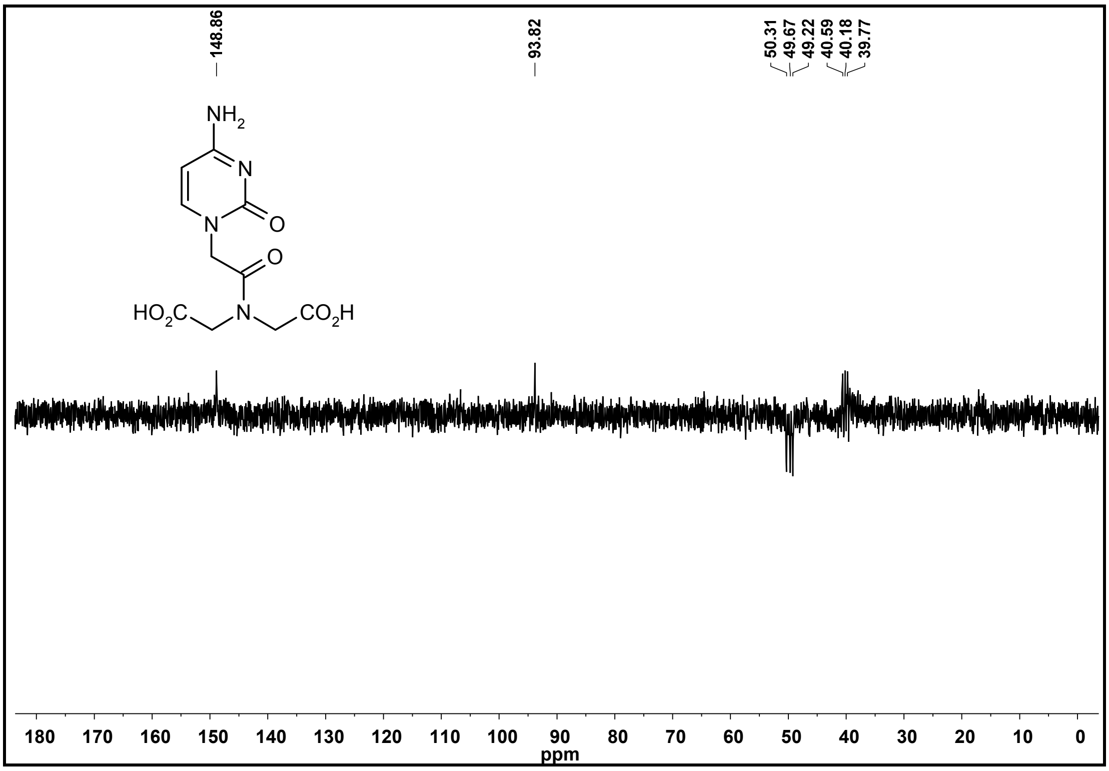

DEPT 135 NMR of compound **21**.

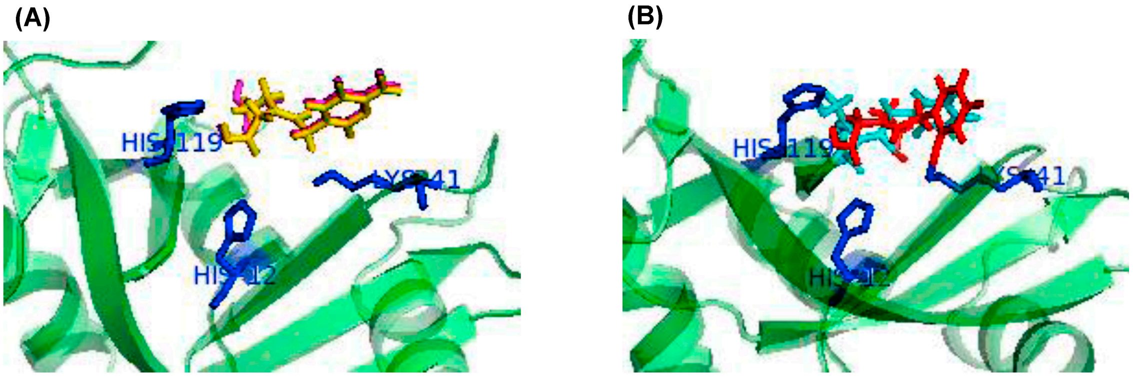

Figure S3. Docked poses of (**A**) **C-di-acid** (**21**) (magenta) and **C-ol-acid** (**18**) (yellow); or (**B**) **U-di-acid** (**10**) (red) and **U-ol-acid** (**8**) (cyan) with RNase A (1FS3).
